# Crocetin: A Systematic Review

**DOI:** 10.3389/fphar.2021.745683

**Published:** 2022-01-14

**Authors:** Zi-Liang Guo, Mao-Xing Li, Xiao-Lin Li, Peng Wang, Wei-Gang Wang, Wei-Ze Du, Zhi-Qiang Yang, Sheng-Fu Chen, Di Wu, Xiu-Yu Tian

**Affiliations:** ^1^ Department of Clinical Pharmacy, The 940th Hospital of Joint Logistic Support Force of Chinese of PLA, Gansu Plateau Pharmaceutical Technology Center, Lanzhou, China; ^2^ College of Pharmacy, Lanzhou University, Lanzhou, China; ^3^ College of Pharmacy, Gansu University of Chinese Medicine, Lanzhou, China; ^4^ Institute of Chemical Technology, Northwest Minzu University, Lanzhou, China; ^5^ College of Pharmacy, Ningxia Medical University, Yinchuan, China

**Keywords:** crocetin, crocetin derivatives, distribution, pharmacological activity, pharmacokinetics, toxicity, formulation

## Abstract

Crocetin is an aglycone of crocin naturally occurring in saffron and produced in biological systems by hydrolysis of crocin as a bioactive metabolite. It is known to exist in several medicinal plants, the desiccative ripe fruit of the cape jasmine belonging to the Rubiaceae family, and stigmas of the saffron plant of the Iridaceae family. According to modern pharmacological investigations, crocetin possesses cardioprotective, hepatoprotective, neuroprotective, antidepressant, antiviral, anticancer, atherosclerotic, antidiabetic, and memory-enhancing properties. Although poor bioavailability hinders therapeutic applications, derivatization and formulation preparation technologies have broadened the application prospects for crocetin. To promote the research and development of crocetin, we summarized the distribution, preparation and production, total synthesis and derivatization technology, pharmacological activity, pharmacokinetics, drug safety, drug formulations, and preparation of crocetin.

## 1 Introduction

**FIGURE 1:**
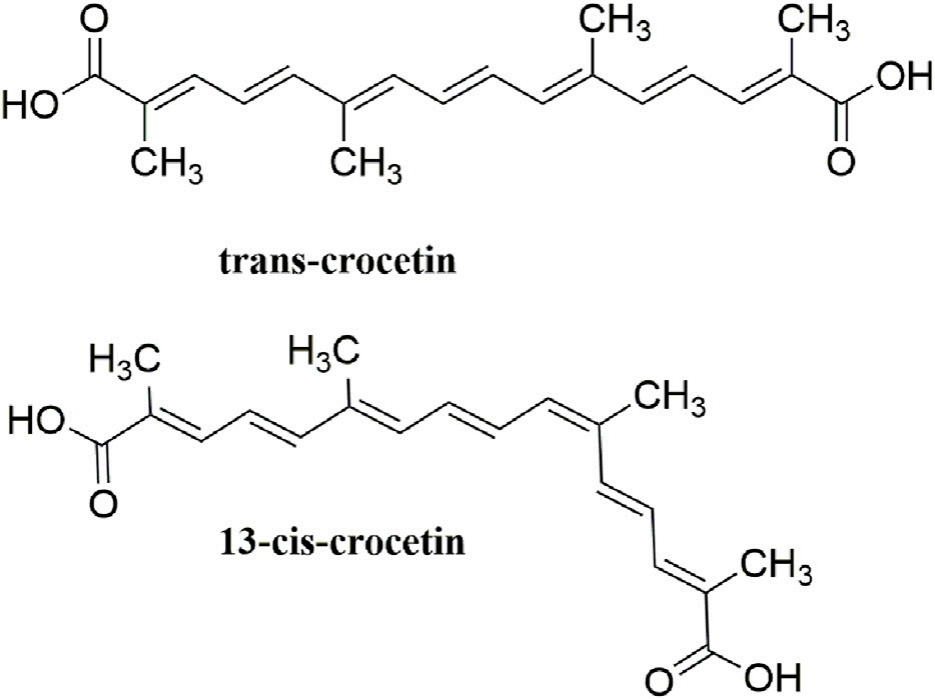
Structural formula of crocetin.

Crocetin is an aglycone of crocin naturally occurring in saffron and is produced in biological systems by hydrolysis of crocin as a bioactive metabolite (Reddy et al., 2020). The structural formula of crocetin is shown in Figure 1. Crocetin (C_20_H_24_O_4_; MW: 328.4 g/mol) displays a polyunsaturated conjugated acid structure, 4 side-chain methyl groups, and seven conjugated double bonds, including *cis*-form and *trans*-form (Peng et al., 2007). Given the presence of a long chain of conjugated carbon-carbon double bonds, crocetin is sensitive to thermal treatment, light, and pH. It undergoes oxidation and isomerization when exposed to light and heat (Na et al.). In addition, it is commonly stabilized by esterification with gentiobiose, glucose, or other common sugar moieties (Moraga et al., 2004). Normally, the *trans*-form is more stable than the *cis*-form. Crocetin exhibits poor solubility in water and most organic solvents, except for pyridine and dimethyl sulfoxide (Eidenberger 2010). Crocetin has been examined using several analytical methods, including high-pressure liquid chromatography (HPLC) and thin-layer chromatography (Sujata et al., 1992). Notably, crocetin has high medicinal value and possesses cardioprotective, hepatoprotective, neuroprotective, antidepressant, antiviral, anticancer, antidiabetic, and memory enhancing properties (Liang and Qian 2006). Crocetin can act *via* different mechanisms, such as enhancing the rate of oxygen transport and diffusivity, inhibiting pro-inflammatory mediators, protecting cells from reactive oxygen species (ROS) damage, and stimulating apoptosis in cancer cells (Mh and Hhb 2019).

This systematic review outlines the distribution, preparation and production, total synthesis and derivatization technology, pharmacological activity, pharmacokinetics, drug safety, drug formulation, and preparation of crocetin, which could provide broad research prospects for exploring and utilizing crocetin.

## 2 Distribution

**FIGURE 2:**
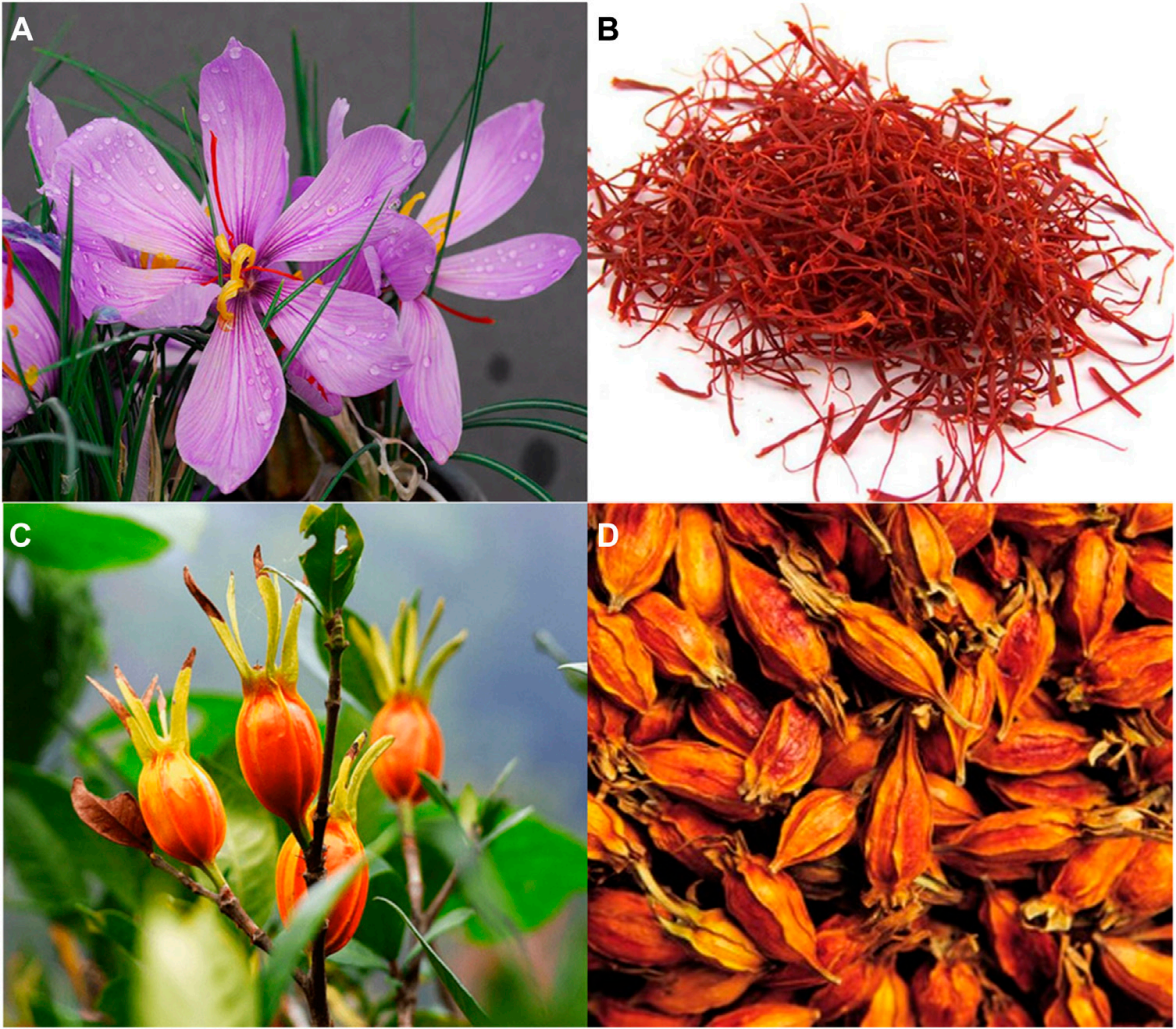
Original plants and medicinal materials of *Crocus sativus* L. and *Gardenia jasminoides* Ellis. **(A)** *C. sativus* L. flower (the picture comes from http://www.plantsoftheworldonline.org/); **(B)** dried stigmas of *C. sativus* L.; **(C)** the fruits of *G. jasminoides* Ellis. (The picture comes from http://www.360doc.com); **(D)** dried fruits of *G. jasminoides* Ellis.

Crocetin is found in *Crocus sativus* L. of Iridaceae, *Gardenia jasminoides* J. Ellis of Rubiaceae (as shown in Figure 2), *Arctium lappa* L. of Asteraceae (Tang et al., 2015), *Stemona japonica* (Blume) Miq. of Stemonaceae (Yang and Tang 2008), *Mimosa pudica* L. of Leguminosae (Patel and Bhutani 2014), *Buddleja officinalis* Maxim. of Loganiaceae (Shi et al., 2016), and *Nyctanthes arbor-tristis* Linn. of Oleaceae (D.Pawar et al., 2015). Among of them, the stigma of *C. sativus* L. and the fruit of *G. jasminoides* J. Ellis contain considerable crocetin (Carmona et al., 2006).

*Crocus sativus* L. originates from Iran, Greece, India, Spain, Morocco and other regions (Cardone et al., 2020). It was first introduced from India to Tibet and named Zanghonghua in China. In addition, it has been artificially cultivated in Zhejiang, Shanghai, Tibet, and other regions in China. Saffron is also known as “red plant gold,” owing to limited resources and low yield (Zhao 2015). Conversely, *G. jasminoides* J. Ellis is widely distributed and cultivated in Jiangxi, Henan, Hubei, Fujian, Sichuan, and other provinces, at a high yield and low cost (Zhang et al., 2013).

## 3 Preparation and Production

Crocetin can be extracted from plant sources using different methods. Saffron, the commercial name of dried stigmas of *C. sativus* L. flowers (Khorasany and Hosseinzadeh 2016), is an extremely expensive spice, given that approximately 80,000 *C. sativus* flowers are required to produce one pound of saffron (Reddy et al., 2020). Therefore, it is cost-ineffective and impossible to extract crocetin from saffron. In contrast, the fruit of *G. jasminoides* J. Eills, which affords a high yield, low cost, and high content of crocetin, is often used as a raw material to extract crocetin for industrial production (Xia et al., 2018).

### 3.1 Preparation of Crocetin From Saffron

Reddy et al. established a method for preparing analytically pure crocetin on a small scale using saffron as raw material. The raw material (*C. sativus* stigma) was sonicated, followed by alkalization and acidification of the supernatant. The resulting precipitate was dissolved in ethyl acetate, and analytically pure crocetin was obtained from ethyl acetate solution (Reddy et al., 2020). In addition, the authors prepared a gram scale for extracting crocetin from saffron raw material. The raw material was extracted with methanol: water, and the obtained extract was hydrolyzed, neutralized, and separated to obtain crocetin (Reddy et al., 2020). Lautenschläger et al. performed enzymatic deglycosylation to extract crocetin from saffron. Two different enzyme preparations were used: RöhmEnzym^®^ and Rohament CL^®^. Further purification was performed using medium pressure liquid chromatography (Lautenschläger et al., 2014).

### 3.2 Preparation of Crocetin From *G. jasminoides* Fruit

Using Amberlite D140 resin chromatography, gardenia yellow pigment was obtained from the 60% ethanol extract of gardenia fruit, which was then alkali-hydrolyzed and acidified. The resulting precipitate was mixed with methanol to remove impurities, and crocetin was crystallized from dimethylformamide (Qian et al., 2010). In another study, the SPE-007A enzyme was selected for enzymolysis of gardenia fruit. After enzymolysis, the obtained materials were alkali-hydrolyzed and then acidified. Crude crocetin was separated using a silica gel column. Finally, crocetin was purified by recrystallization (Zhang W. et al., 2017).

### 3.3 Bioengineering

Tan et al. studied the effect of a specific aldehyde dehydrogenase (*CsALDH3*) on the oxidation of crocetin dialdehyde to crocetin. The authors predicted that four *CsALDH* genes encode enzymes responsible for catalyzing crocetin dialdehyde conversion to yield crocetin. To characterize the function of candidate *CsALDH* genes, nucleotide sequence analysis was performed to identify the full-length transcripts. Accordingly, three cDNAs (*CsALDH1, CsALDH2, and CsALDH3*) were predicted as candidate genes involved in crocetin biosynthesis. Codon-optimized *CsALDHs* were individually introduced into the zeaxanthin-producing yeast. Expression of the recombinant *CsALDH3* protein in crocetin-producing yeast strains resulted ina39% increased yield (Tan et al., 2019). Song et al. optimized the overproduction of crocetin in yeast. By blocking genes related to citric acid synthase (*CIT2*) in the glyoxylate cycle, the crocetin titer could be elevated by 50% when compared with the starting strain. Accordingly, the crocetin yield was further elevated by 44% by introducing the forward fusion enzyme *Ps*CrtZ-*Cs*CCD2. Finally, the crocetin titer was 12.43 ± 0.62 mg/L in a 5 L bioreactor (Song et al., 2020). In addition, the resulting engineered strain was characterized by overexpression of *CrtZ* and *CCD* genes. The engineered strain displayed higher efficiency in crocetin production, and the concentration of crocetin reached 1.17 mg/L after fermentation for 108 h (Xiao et al., 2019). Lou et al. introduced a plant expression vector carrying *crtRB* and *ZCD1* genes into *C. vulgaris*; *crtRB* and *ZCD1* genes encode key enzymes that control crocetin biosynthesis. Crocetin can be produced in transgenic *C. vulgaris* but not in the wild-type species (Lou et al., 2016).

Obviously, *G. jasminoides* fruit is more cost-effective than saffron for crocetin production. In addition, alkali hydrolysis is simple and easy, and enzymolysis is considered more eco-friendly than other methods. In the 21st century, bioengineering can broaden prospective resources for crocetin extraction and production.

## 4 Total Synthesis and Derivatization

Structural modification is expected to improve the solubility, bioavailability, and pharmacological activity of crocetin, potentially expanding the application of crocetin (Yang 2012) (Table 1).

**TABLE 1:** Structural formula of crocetin derivatization.

No	Name	Structure	References
1	Crocin-1	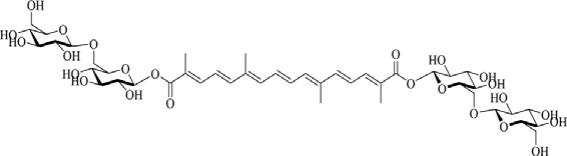	Ding et al. (2018)
	Crocin-2	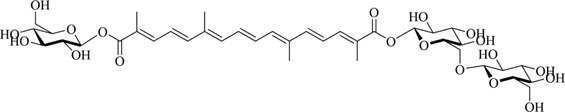	
	Crocin-3	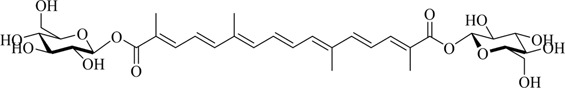	
	Crocin-4	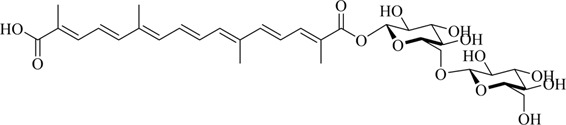	
	Crocin-5	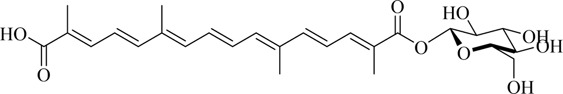	
2	Crocetin diammonium salt	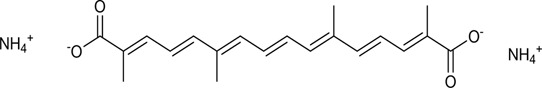	Yang (2012)
3	Crocetin dialdehyde	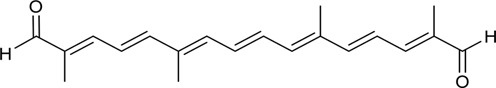	Zhang and Luo (2016)
4	Crocetin sodium	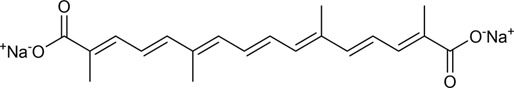	Zhang (2017)
5	Crocetin dimethyl ester	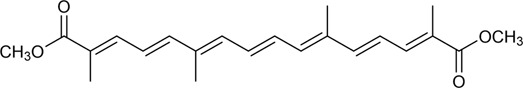	Fang and Wang (2007)
6	Crocetin amide derivatives	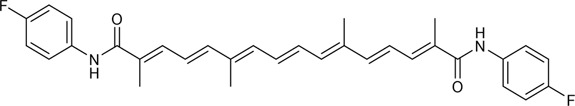	Wang et al. (2020a)
7	Crocetin amide derivatives	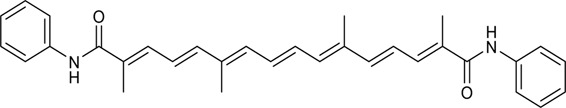	Zhu et al. (2012)
8	Crocetin organic amine salt	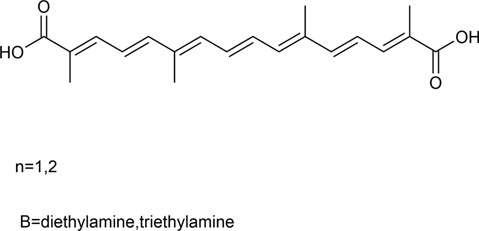	Yang et al. (2011)

### 4.1 Crocetin

Fang et al. applied for a patent on crocetin synthesis *via* organic chemistry. The method used 3,7-dimethyloctatrienemthanal and methyl 2-bromopropionate as raw materials to synthesize crude crocetin as a dimethyl ester *via* a three-step reaction, the refined crocetin was obtained after hydrolysis, decoloration, and recrystallization (Fang and Wang 2007).

### 4.2 Crocin

Microbial glycosyltransferases [GTs; bacterial GTs (Bs-GT)] extracted from *Bacillus subtilis* 168 by Ding et al. showed a high degree of carboxyl glycosylation activation for crocetin. The molecular conversion rate approached 81.9%, affording 476.8 mg/L crocin, thus indicating the efficient production of crocin. Rare crocin-5 and crocin-3 are specifically produced by Bs-GT (Ding et al., 2018).

### 4.3 Crocetin Diammonium Salt

Yang et al. applied for a patent for the preparation of crocetin diammonium salt. Crocetin diammonium salt was extracted from *G. jasminoides* with ammonia water and concentrated to a thick paste by adding organic solvents (methanol, isopropanol). The diammonium salt of crocetin was completely precipitated after chilling (owing to the low solubility of the diammonium salt of crocetin in organic solvent). The crude product of the diammonium crocetin salt was obtained by filtration. Then, HPD-100 resin was used to separate the diammonium salt of crocetin (Yang 2012).

### 4.4 Crocetin Dialdehyde

Crocetin dialdehyde was synthesized by reacting 2,7-dimethylocta-2,4,6-trienedial with diethyl 3-(5,5-dimethyl-1,3- dioxane-2-yl) but 2-enylphosphonate *via* the Horner-Wadsworth-Emmons reaction. This method yielded a 41% crocetin dialdehyde (Zhang and Luo 2016).

### 4.5 Crocetin Sodium

Purified crocetin was added to a sodium hydroxide solution at a molar ratio of 1:2. After the reaction was complete, crocetin sodium salt was obtained by filtration, sterilization, and freeze-drying. The total crocetin yield was 1.15% (Zhang 2017).

### 4.6 Crocetin Dimethyl Ester

The gardenia yellow pigment was added to anhydrous methanol and a sodium methoxide solution. After changing to an ester, crocetin dimethyl ester was obtained. The purity of crocetin dimethyl ester was 98.8% by recrystallization (Fang and Wang 2007). In the synthesis experiment designed by Sun et al., crocetin dimethyl ester was obtained using the Wittig reaction, combining 2,7-dimethyl-2,4,6-octatriene-1,8- dialdehyde and γ-chloro methyl tiglate to achieve a crocetin dimethyl ester yield of 78.6%. Of these reagents, 2,7-dimethyl-2,4,6-octatriene-1,8- dialdehyde was synthesized *via* the Wittig-Horner reaction using dimethoxyacetone and 1,4-dibromo-2-butene as raw materials; γ-chloro methyl tiglate was synthesized from chloroacetaldehyde and 2-bromo methyl propionate (Sun et al., 2012).

### 4.7 Crocetin Amide Derivatives

Crocetin was mixed and reacted with oxalyl chloride and triethylamine, followed by the addition of phenylethylamine. The reaction solution was extracted with an organic solvent, and crocetin amide derivatives were obtained by recrystallization (Zhu et al., 2012). In another method designed by Wang et al., crocetin was added to HOBt and EDCl, followed by Et3N and 4-fluorobenzylamine. Synthetic crocetin derivatives were acquired by vacuum evaporation, and purified crocetin derivatives were obtained by column chromatography. After structural modification, the formation of hydrogen bonds increased, along with the solubility of obtained crocetin derivatives (Wang MZ. et al., 2020).

### 4.8 Crocetin Organic Amine Salt

Dimethylformamide and organic amine were added to crocetin as the reaction solution, followed by ethyl acetate and petroleum ether. Organic amine salt crystals were obtained by precipitation, filtration, and recrystallization (Yang et al., 2011).

### 4.9 Crocetin Glucose Ester

GTs can specifically transfer sugar groups to receptor molecules (Modenutti et al., 2019). He et al. applied to patent the preparation of crocetin glucose ester using glucose as the donor. GT from *B. subtilis* was used as a glycosyl donor to synthesize crocetin glucose ester. Crocetin and UDP-Glc were added to a phosphate buffer solution or glycine NaOH buffer solution to perform the reactions (He et al., 2017).

Overall, salinization and esterification are the main derivative strategies. However, comparisons examining the pharmacokinetics, bioavailability, and pharmacological activities of these derivatizations were insufficient.

## 5 Pharmacological Activities

**FIGURE 3:**
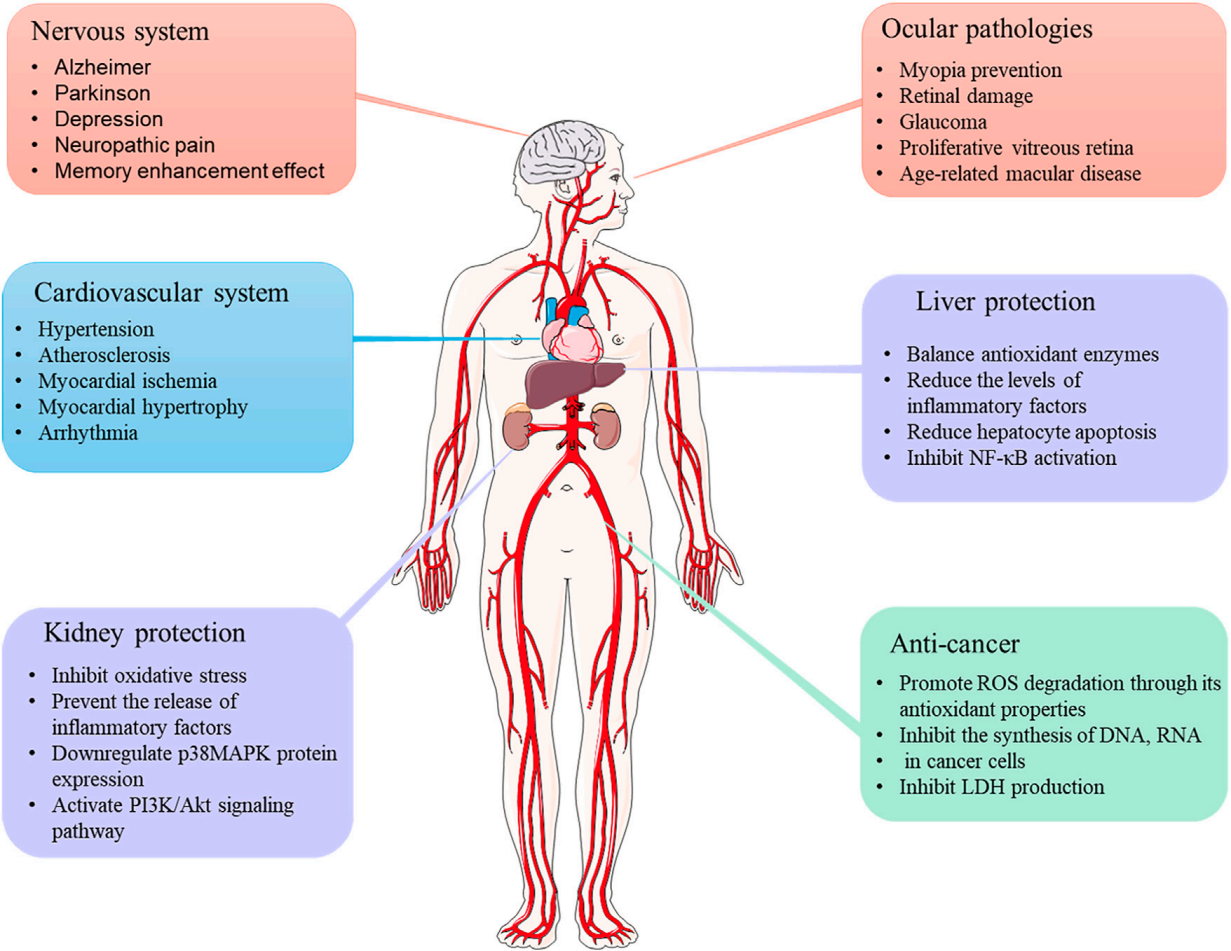
Therapeutic applications of crocetin.

It has been reported that crocetin mediates the therapeutic properties of saffron (Fernández-Albarral et al., 2020). Crocetin exhibits various pharmacological effects, including cardioprotective, hepatoprotective, neuroprotective, antinociceptive, antidepressant, antiviral, anticancer, atherosclerotic, antidiabetic, and memory enhancer properties. Studies assessing the pharmacological activities of crocetin are discussed in detail below (Figure 3).

### 5.1 Cardiovascular System

Studies have shown that crocetin plays a potential role in prevention and treatment of cardiovascular diseases such as hypertension, myocardial hypertrophy, myocardial ischemia, atherosclerosis (Table 2).

**TABLE 2:** Effect of crocetin on cardiovascular diseases.

Pharmacologic action	Subjects	Doses	Mechanism of action	References
Hypertension	Spontaneously hypertensive rats	Crocetin (1.2 × 10^–5^ M)	Induces vasodilation *via* endothelial nitric oxide pathway	Mancini et al. (2014)
	Hereditary hypertension rats		Endothelium-dependent relaxation promoting effect	Llorens et al. (2015)
	Stroke-prone spontaneously hypertensive rats		Reduces oxidative stress induced by ROS	Yoshino et al. (2011)
	Stroke-prone spontaneously hypertensive rats	Crocetin (50 mg/kg)	Reduces the inactivation of NO-induced by ROS	Higashino et al. (2014)
Myocardial hypertrophy	Cardiac hypertrophy mice	Crocetin (50 mg/kg)	Blocks MAPK and MEK/ERK1/2 pathways	Cai et al. (2009)
	Myocardial hypertrophy rats		Decreases the LPO content and increases the activities of GSH-Px and SOD	Shen and Qian (2006)
Myocardial ischemia	Acute myocardial ischemia rats		Reduces the release of CK and LDH	Liu and Qian (2002)
	Myocardial ischemia-reperfusion injury rats	Crocetin derivative (9 mg/kg)	Reduces oxidative stress injury and expression of inflammatory factors	Liu (2019)
	H9c2 cardiomyocytes	Crocetin derivative (10 μmol/L)	Ameliorates the apoptosis of cardiomyocytes and reduces the expression level of intracellular ROS	Liu (2019)
	Myocardial ischemia-reperfusion injury rats	Crocetin (50 mg/kg)	Decreases the levels of CK-MB, TNF-α, and MDA increases the activities of T-SOD and IL-10	Wang et al. (2014)
Arrhythmia	Antiarrhythmic rats and guinea pigs		Inhibit Na^+^ influx or Ca^2+^influx	Cheng et al. (2010)
	HEK-293 cells	Crocetin (1, 3, 10, 30 μmol·L^−1^)	No significant effect on the expression of HERG potassium channel protein	Wang and Shen (2012)
Myocardial infarction	Myocardial infarction rats	Crocetin (50, 100, 200 mg/kg/day)	Enhances the expression of Bcl-2 by reducing the levels of caspase-3, Bax, and Nrf-2	Zhang et al. (2017a)
Cardiac insufficiency	H9c2 cells		Increases Bcl-2 activity and PI3K-Akt signaling pathway, upregulates the expression levels of Nrf2, HO-1, and NQO1, maintains mitochondrial function	Wang et al. (2020b)
Atherosclerosis	Atherosclerosis rats	Crocetin (25, 50 mg/kg)	Downregulates the expression levels of the LOX-1 gene and protein	Cai et al. (2012)
	Hyperlipidemia rabbits	Crocetin (30 mg/kg)	Reduces serum TG, TC, LDL-C levels	Zheng et al. (2009)
	Atherosclerosis rats	Crocetin (25, 50 mg/kg)	Downregulates the p38 MAPK pathway	Diao et al. (2018)
	50 patients with CAD	Crocetin (10 mg)	Change the expression of endothelial cell adhesion molecules and atherogenic genes	Abedimanesh et al. (2019)
Antithrombosis		Crocetin (25, 50 mg/kg)	Inhibit intracellular Ca^2+^ release and extracellular Ca^2+^ influx	Yang et al. (2008)
	DIC rabbits	Crocetin (3 mg/kg)	Improves the DIC-related hemostatic indices	Tsantarliotou et al. (2013)
Angiogenesis	HUVECs	Crocetin (1, 5, 25, 50, 100 μmol/L)	Activates PI3K-Akt-eNOS signaling pathway	Mahdieh et al. (2019)
Protective effect of diabetic vascular disease	Diabetic rats	Crocetin (50 mg/kg/day)	AGE deposition and RAGE expression are decreased	Xiang et al. (2006)

#### 5.1.1 Hypertension

Mannich et al. analyzed the effect of crocetin on vascular regulation during hypertension. Acetylcholine (ACH)-induced spontaneously hypertensive rats (SHRs) were used as disease models. Crocetin (1.2 × 10^−5^M) increased aortic ACH relaxation in SHRs. Considering the underlying mechanism, crocetin induced vasodilation *via* the endothelial nitric oxide (NO) pathway. Dietary supplementation with crocetin may be a good strategy for treating hypertension (Mancini et al., 2014). Llorens et al. studied the regulatory effects of crocetin and crocin on smooth muscle contraction in hereditary hypertension. These authors suggest that crocetin (1.2 × 10^–5^ M) promotes endothelium-dependent relaxation, and crocin has antihypertensive activity (Llorens et al., 2015). Higashino et al. administered crocetin (25 and 50 mg/kg/day) to stroke-prone SHRs for 3 weeks by oral administration. Crocetin significantly inhibited the increase in systolic blood pressure, as well as significantly reduced thrombogenesis in pial vessels. After treatment with crocetin, the levels of both urinary 8-hydroxy-2′-deoxyguanosine and nitroxide metabolite (NO_2_/NO_3_) were elevated, indicating that the antioxidant activity was significantly increased. This mechanism may be mediated by reducing the ROS-induced NO inactivation (Higashino et al., 2014).

#### 5.1.2 Myocardial Hypertrophy

Crocetin suspension (50 mg/kg) was administered to animal models of cardiac hypertrophy *via* intragastric administration thrice daily for 1 week. Crocetin reversed myocardial hypertrophy *in vivo*, possibly by blocking the reactive oxygen species-dependent mitogen-activated protein kinase (MAPK)/extracellular signal-regulated kinase-1/2 (MEK/ERK1/2) pathway, thus protecting mice from the harmful effects of myocardial hypertrophy, fibrosis, and inflammation (Cai et al., 2009). An experiment assessing the protective effect of crocetin on norepinephrine (NE)-induced myocardial hypertrophy in rats revealed that crocetin significantly decreased the lipid peroxidation (LPO) content and increased the activities of glutathione peroxidase (GSH-Px) and superoxide peroxidase (SOD) in myocardial hypertrophy tissue. Cellular image analysis indicated that crocetin improved pathological histological changes observed in NE-induced myocardial hypertrophy (Shen and Qian 2006).

#### 5.1.3 Myocardial Ischemia

Liu et al. reported that crocetin (25, 50, 100 mg/kg) significantly reduced the release of creatine kinase (CK) and lactate dehydrogenase (LDH) in serum, as well as serum malondialdehyde (MDA) levels and myocardial homogenate of acute myocardial ischemia model rats induced by ISO (Liu and Qian 2002). Ischemia-reperfusion (I/R) injury refers to the phenomenon in which reperfusion after ischemia fails to restore tissue and organ functions in humans and animals but further aggravates the ischemia-induced functional metabolic disorder and structural damage (Murphy and Steenbergen 2008). Crocetin (25, 50 mg/kg) has a protective effect on myocardial I/R injury in rats by boosting ATPase activities associated with energy metabolism (Wen et al., 2005). Liu et al. examined the effect of crocetin derivative (GX) on myocardial I/R injury in rats and cardiomyocytes. In H9c2 cardiomyocytes, GX (10 μmol/L) significantly improved the cell survival rate, ameliorated cardiomyocyte apoptosis, and reduced the expression levels of intracellular ROS in the hypoxia/reoxygenation injury model induced by hyposulfurous acid. Intravenous GX (9 mg/kg) significantly reduced the infarct size and myocardial ischemic area in myocardial I/R injury rats. In addition, GX reduced the activities of CK and LDH in rat plasma and inhibited the gene expression of inflammatory factors tumor necrosis factor (TNF)-α, interleukin (IL)-6, and IL-1β in plasma. These results suggested that crocetin may afford protection against myocardial I/R injury by reducing oxidative stress injury and inflammatory factor expression in myocardial tissues (Liu 2019); additional studies have also demonstrated this effect. Rats with myocardial I/R injury were pretreated with crocetin (50 mg/kg/day) for 7 days by intragastric administration. The myocardial infarct area was significantly reduced. The myocardial tissue levels of CK- myocardial band (MB), TNF-α, and MDA were decreased, and the activities of total SOD (T-SOD) and IL-10 were increased. Moreover, crocetin reduced Bax expression and enhanced Bcl-2 expression, suggesting that crocetin inhibited apoptosis (Wang et al., 2014).

#### 5.1.4 Arrhythmia

Cheng et al. used rats and guinea pigs as experimental animals. Rapid injection of 4% calcium chloride is known to induce arrhythmia in animal models. The duration of ventricular premature contraction (VE), ventricular fibrillation (VF), and cardiac arrest were examined. The authors revealed that crocetin significantly reduced mortality, as well as the incidence of premature VE and VF induced by calcium chloride in rats. The antiarrhythmic effect of crocetin might be related to the inhibition of Na^+^ or Ca^2+^ influx (Cheng et al., 2010). Zhao et al. found that crocetin (600 μg/ml) decreased L-type Ca^2+^ currents (ICa-L; 35.56 ± 2.42%) in ischemic myocytes and abated the crest value of the ephemeral Ca^2+^ by 31.87 ± 2.57%. The time to half-peak for Ca^2+^ and time constant of the transient decay were both reduced. These findings revealed the potential effect of crocetin as a calcium channel antagonist for treating cardiovascular diseases (Zhao Y. J. et al., 2020). The effects of crocetin (1, 3, 10, and 30 μmol·L^−1^) on the human ether-a-go-go-related gene (hERG) potassium channel protein expression were examined in HEK-293 cells. The results demonstrated that crocetin had no significant effect on the expression of HERG potassium channel protein, excluding its inhibitory effect on the expression of HERG potassium channel protein, which may result in QT prolongation. This study provided theoretical support indicating the safety of crocetin for treating arrhythmia from the perspective of molecular biology (Wang and Shen 2012).

#### 5.1.5 Myocardial Infarction

Zhang et al. established myocardial infarction in rats by administering an intravenous infusion of isoproterenol to assess the protective effect of crocetin on myocardial injury. The rats were orally administered crocetin (50, 100, and 200 mg/kg/day) for 15 days. The results showed that the oxidative stress indexes such as GSH and catalase (CAT) levels in the crocetin treatment group were elevated, whereas MDA and SOD activities were reduced. Moreover, the levels of inflammatory factors in the myocardial cells were reduced. The decrease in serum myocardial enzymes (LDH, CK-MB) also indicated that crocetin exerted a certain therapeutic effect on acute myocardial infarction. In addition, crocetin inhibited cardiomyocyte apoptosis, which mainly enhanced the expression of the anti-apoptotic protein Bcl-2 by reducing the levels of caspase-3, Bax, and nuclear factor-erythroid factor 2-related factor 2 (Nrf-2) (Zhang Y. L. et al., 2017).

#### 5.1.6 Cardiac Insufficiency

The effect of crocetin on sepsis-induced cardiac dysfunction was evaluated. Lipopolysaccharide (LPS)-induced H9c2 cells induced were used as an *in vitro* model of cardiac sepsis. The results revealed that crocetin (50 mmol) alleviated myocardial toxicity in an LPS-induced sepsis model by upregulating SOD and GSH-Px expression and decreasing the MDA content. Crocetin significantly alleviated LPS-induced cellular apoptosis by increasing Bcl-2 activity and the PI3K-Akt signaling pathway. Crocetin regulated the inflammatory response of cardiomyocytes and significantly upregulated the levels of Nrf2, heme oxygenase (HO-1), and NAD(P)H:quinone oxidoreductase (NQO1). Treatment with crocetin protected mitochondrial respiration, prevented mitochondrial fragmentation, and suppressed changes in LPS-induced mitochondrial fusion and fission protein expression levels. In summary, the results suggest that crocetin potentially reduces sepsis-induced cardiac dysfunction by reducing cytotoxicity, apoptosis, mitochondrial dysfunction, and inflammation, thus facilitating the maintenance of normal cardiomyocyte function (Wang Y. et al., 2020).

#### 5.1.7 Atherosclerosis

Reportedly, crocetin effectively inhibited the proliferation of vascular smooth muscle cells (VSMCs) induced by platelet-derived factor (PDGF-BB), downregulated the over-activation of the PI3K/Akt pathway, and exhibited anti-atherosclerotic potential (Zhang et al., 2018). According to the theory of oxidative stress, modification of low-density lipoprotein (LDL) oxidation is a crucial link in the formation and development of atherosclerosis. When LDL is oxidized to Ox-LDL, the receptor binding site is altered, and this change is not negatively regulated by the intracellular cholesterol content. Combined with Ox-LDL, lipoprotein receptor-1 (LOX-1) can activate extracellular signal-regulating kinases (ERK), induce VSMCs to migrate to the intima, promote smooth muscle cell proliferation, and increase lipid intake, thus resulting in pathological vascular changes, eventually leading to the occurrence of vascular diseases such as atherosclerosis. Crocetin (25, 50 mg/kg, for 12 weeks by i.g.) was shown to significantly downregulate the expression levels of the LOX-1 gene and protein in atherosclerotic rats (Cai et al., 2012). Based on the theory of lipid metabolism disorder, hyperlipidemia is the main risk factor for atherosclerosis, and the deposition of plasma lipids in the vascular wall remains the main underlying mechanism (Drechsler et al., 2010). Several experiments using different animal models have shown that oral administration of crocetin (5–50 mg/kg) reduced serum triacylglycerol (TG), total cholesterol (TC), LDL, and very-low-density lipoprotein levels *via* antioxidant and anti-inflammatory effects associated with the p38 MAPK pathway (Zheng et al., 2009; Diao et al., 2018; Yu et al., 2021).

In a clinical trial assessing the effect of crocetin for treating atherosclerosis, 50 patients diagnosed with coronary artery disease (CAD) were randomly divided into two groups, i.e., crocetin and placebo, to receive one capsule of crocetin (10 mg) and placebo, respectively, once daily for 60 days. Compared with the placebo group, the crocetin group showed significantly increased serum homocysteine (Hcy) and heart-type fatty acid-binding protein (h-FABP). In addition, the gene expression of sirtuin1 and AMP-activated protein kinase was increased, while the expression levels of oxidized LDL receptor 1 and nuclear factor-kappa B (NF-κB) were decreased in isolated peripheral blood mononuclear cells in the crocetin group. Accordingly, crocetin could alter the expression of endothelial cell adhesion molecules and atherogenic genes in patients with CAD (Abedimanesh et al., 2019). Angiotensin II (Ang II) is a factor known to induce vascular smooth muscle proliferation (Xu 2019). Studies have shown that crocetin can inhibit ERK 1/2 phosphorylation and activation induced by Ang II, thereby inhibiting the proliferation of VSMCs (Zhou et al., 2006). These findings suggest that one possible mechanism through which crocetin alleviates atherosclerosis might involve the inhibition of VSMC proliferation. Crocetin (0.01, 0.1, and 1 μmol) suppressed the expression of cyclin D1 and elevated the level of cyclin-dependent kinase inhibitor p27kip1 (CDKIp27kip1), decreasing the proportion of VSMCs in the S-phase and increasing the VSMC proportion in the G0/G1 phase when compared with Ang-II-induced VSMCs (Zhou et al., 2010).

#### 5.1.8 Myocarditis

Qin et al. used a coxsackievirus B3 (CVB3)-induced myocarditis mouse model to determine whether crocetin afforded cardioprotective effects in a model of acute viral myocarditis. Crocetin (2.5, 5 mg/kg) was injected intraperitoneally for 14 days. The authors revealed that crocetin treatment improved the survival rate of CVB3-infected mice and alleviated myocardial necrosis, decreased the levels of IL-6, IL-1β, and TNF-α, and reduced CVB3 replication and IL-17 expression in the infected hearts (Qin et al., 2021).

#### 5.1.9 Antithrombosis

Yang et al. examined the effect of crocetin on platelet activity and thrombosis. The authors showed that crocetin (25 and 50 mg/kg) reduced collagen-induced platelet aggregation in rats, with inhibition ratios of 36.6 and 33.3%, respectively. The antiplatelet activity of crocetin might be related to the inhibition of intracellular Ca^2+^ release and extracellular Ca^2+^ influx. In addition, crocetin prolonged the occlusion time of carotid artery thrombosis, which was induced by electrical stimulation (Yang et al., 2008). In a study by Tsantarliotou et al., bacterial endotoxin-induced disseminated intravascular coagulation (DIC) in rabbits was used to assess the effect of crocetin on thrombosis. Prior to the endotoxin injection, models were administered crocetin (3 mg/kg), which improved the DIC-related hemostatic indices, such as plasma fibrinogen, platelet count, and protein C concentration, and ameliorated fibrin deposition in the glomeruli (Tsantarliotou et al., 2013).

#### 5.1.10 Angiogenesis

One strategy to alleviate ischemia and tissue healing is the facilitation of angiogenesis. Mahdieh et al. revealed that crocetin could promote angiogenesis in human umbilical vein endothelial cells (HUVECs) *via* the PI3K-Akt-ENOS signaling pathway. Incubation with different crocetin concentrations for 72 h (1, 5, 25, 50, and 100 μmol/L) increased the viability and proliferation of HUVECs and promoted the formation of capillary-like structures. Crocetin increased the activity of matrix metalloproteinase (MMP-9) in HUVECs and enhanced the uptake of acetylated-LDL (Ac-LDL). Treatment with crocetin increased the ratio of vascular endothelial growth factor receptor (VEGFR)-1, -2, p-Akt/Akt, and phospho endothelial NO synthase (p-eNOS)/eNOS in HUVECs. However, crocetin reduced VEGF transcription. In conclusion, crocetin promoted the angiogenesis potential of HUVECs by regulating the VEGF signaling pathway and improving cell viability (Mahdieh et al., 2019).

#### 5.1.11 Stroke

Yoshino et al. used electron spin resonance and spin-trapping techniques to demonstrate the antioxidant effect of crocetin. Electron spin resonance analysis revealed that crocetin significantly reduced oxidative stress in isolated brains of stroke-prone SHRs, indicating that crocetin could prevent ROS-related brain diseases, such as stroke (Yoshino et al., 2011). Liu et al. established a rat model of middle cerebral artery occlusion to simulate ischemic stroke *in vivo* and used human U87 glioma cells with oxygen and glucose deprivation to simulate cerebral ischemia. Crocetin [50 mg/kg (p.o.)] treatment ameliorated the infarct volume and pathological status *in vivo*. *In vitro*, the apoptosis rates decreased with crocetin (50 mg/L) treatment. The underlying mechanism could be related to the regulation of the miR145-5p/TLR4 axis (Liu et al., 2021).

#### 5.1.12 Shock

Yan et al. demonstrated that crocetin (50 mg/kg) could improve cardiac damage caused by hemorrhagic shock and resuscitation in rats due to blocking inflammatory factors, inhibiting ROS production, and preserving T-SOD activity (Yan et al., 2010).

#### 5.1.13 Hyperlipidemia

Crocetin [50 mg/kg (p.o.)] can inhibit pancreatic lipase activity and reduce TC and TG levels (Lee et al., 2005). Likewise, 4T1-induced breast cancer mice were intraperitoneally administrated crocetin (150 mg/kg), once a week, for 4 weeks. The results demonstrated that crocetin reduced TC and TG levels in cancer tissues and serum from breast cancer mice. (Hashemi et al., 2020).

### 5.2 Anti-Cancer

Several theories exist regarding cancer occurrence, and the theory of “oxidative stress” is worthy of further attention (Sosa et al., 2012). Higher ROS levels in cancer cells have been found and used to explain the mechanisms of tumor growth, proliferation, and metastasis. Numerous studies have demonstrated the anticancer effects of crocin, crocetin, and other anticancer agents *via* the regulation of antioxidant activity, reduced cyclooxygenase (COX)-2 production and inflammation, induction of cell apoptosis, and antiproliferative activity (Hashemi S. et al., 2018; Zou et al., 2017). Studies have shown that crocetin can inhibit the synthesis of DNA, RNA, and proteins in cancer cells (Colapietro et al., 2019). Azarhazin et al. confirmed that crocetin, as an anticancer drug, interacted with Dickerson DNA through van der Waals forces and hydrogen bonds, and the active site was found to be located in the small groove of DNA (Azarhazin et al., 2017). In addition, crocetin reportedly influences the growth of cancer cells by blocking the growth factor signaling pathway, arresting the cell cycle, and inducing apoptosis (Gutheil et al., 2012).

*In vivo* and *in vitro* experiments have revealed that crocetin has therapeutic effects against breast, skin, gastrointestinal, liver, cervical, and ovarian cancers (Colapietro et al., 2019; Hashemi and Hosseinzadeh 2019) (Table 3).

**TABLE 3:** Anti-cancer effect of crocetin.

Pharmacologic action	Subjects	Doses	Mechanism of action	References
Breast cancer	Breast cancer rats		Reduces the number of tumors	Maysam et al. (2011)
	Breast tumor BALB/c mice	Crocetin (100 mg/kg)	Overexpresses EcSOD and increases antioxidant activity	Sah et al. (2020)
	MCF-7 cells	Crocetin (200 μmol/L)	Inhibits SOD activity by affecting copper binding sites	Sah et al. (2020)
	MCF-7 cells	Crocetin glucosyl ester IC_50_ from 31.25 to 1,000 μg/ml	Inhibits estrogen receptor α and HDAC2 mediated signaling cascade	Mam et al. (2020)
Esophageal cancer	KYSE-150 cells	Crocetin (0, 12.5, 25, 50, 100, 200 μmol/L)	S-phase cell arrest	Li et al. (2015)
	KYSE-150 cells	Crocetin (200 μmol/L)	Upregulates the p53/p21 pathway	Li et al. (2017)
Gastric cancer	AGS cells	Crocetin (50–240 μmol/L)	Decreases the Bcl-2/Bax ratio of AGS cells	Bathaie et al. (2013a)
	Gastric cancer rats		Reverses changes in serum antioxidant activity and LDH in rats	Bathaie et al. (2013b)
	SGC7901 cells	Crocetin (12.5, 25, 50 μmol/L)	Upregulates Bax proteins expression and downregulates Bcl-2 protein expression	Zhang (2020)
Colon cancer	HCT116 cells	Crocetin (30 µM)	Downregulates inflammation-related genes, HMGB1, IL-6, and IL-8	Zhuang et al. (2018)
	SW480 cells	Crocetin (0.8 mM/L)	Activates p21 in a P53- independent manner	Li et al. (2012)
	P53 damage cancer cells		Exploits P73 (P53 paralog) through the FAS-associated death domain to induce apoptosis of colon cancer	Ray et al. (2016)
Pancreatic cancer	Cancer stem cells (CSC)		Inhibits the expression of Sonic hedgehog (SHH)	Parthasarathy et al. (2015)
	MIA-PaCa-2 cells	Crocetin (50, 100, 200 μmol/L)	Enhances Cdc-2 phosphorylation and inhibits Cyclin B1	Dhar et al. (2009)
	Mice without thymus are injected with MIA-PaCa-2 cells	Crocetin (4 mg/kg/day)	Increases the Bax/Bcl-2 ratio	Dhar et al. (2009)
Cervical cancer	HeLa cells	(240 μmol/L)	Inhibits the proliferation of cancer cells by inducing cell cycle arrest at the G1 phase	(Zhong et al., 2011)
	Cervical cancer model in mice		Attenuates the serum levels of IL-1β, TNF-α, PMN, and nitrates	Chen et al. (2015)
	HeLa cells		Upregulates COX-2 expression	Chen et al. (2015)
Lung cancer	Lung cancer animal	Crocetin (20 mg/kg)	Increases the activities of glutathione metabolic enzymes and antioxidant enzymes	Magesh et al. (2006)
	Lung cancer mice	Crocetin (50 mg/kg)	Inhibits polyamine synthesis and glycoprotein changes	Magesh et al. (2010)
	A549 cell	Crocetin disodium salt	Inhibits LDH	Granchi et al. (2017)
Prostate cancer	Two invasive PCa cell lines (PC3 and 22rv1) in male nude mice	Crocetin (30 mg/kg)	Interferes with topoisomerase II to induce DNA damage and apoptosis, inhibits the migration and invasion of PCa cells	Claudio et al. (2014)
Ovarian cancer	A2780 cells	Crocetin (25, 50, 100, 200 μmol/L)	Reduces the gene expression and efflux function of MRP2 transporters	Neyshaburinezhad et al. (2019)
Leukemia	HL-60 cells		Inhibit cells proliferation and differentiation	Tarantilis et al. (1994)
	APL cells, NB4 and HL60 cells	Crocetin (100 μmol/L)	Reduces the expression of prosurvival genes and multidrug resistance proteins and inhibits tyrosyl DNA phosphodiesterase 1	Maliheh et al. (2019)
Skin cancer	Female CD-I mice	Crocetin (0.2 or 1.0 μmol/L)	Inhibits the production of myeloperoxidase and hydrogen peroxide	Wang et al. (1995)
	B16F10 murine melanoma cells		Reduces protein levels of tyrosinase and microphthalmia-associated transcription factor	Hashemi et al. (2018)

#### 5.2.1 Breast Cancer

Crocin and crocetin were administered to N-methyl-nitrosourea (NMU)-induced breast cancer in rats. Palpation results revealed that tumors were significantly reduced in the treatment group (Maysam et al., 2011). *In vitro*, crocin and crocetin showed intense SOD inhibition and radical scavenging activity in MCF-7 breast cancer cells. Based on docking data of crocin and crocetin with SOD crystal structure, crocin/crocetin exhibited distinct SOD binding sites. Crocin inhibited SOD activity by scavenging superoxide free radicals (O_2_•), whereas crocetin inhibited this activity by affecting the copper binding sites. However, *in vivo*, both crocin and crocetin effectively improved SOD activity in BALB/c mice after 1 month of treatment, possibly due to the overexpression of extracellular SOD (EcSOD) and increased antioxidant activity (Hashemi-Shahri et al., 2018). In another report, crocetin β-D glucosyl ester inhibited proliferation in MCF-7 cells in a dose-dependent manner, with an IC_50_ value of 628.36 mg/ml. However, crocetin had no significant effect on the normal cell line (L-6). Crocetin β-D glucosyl ester exerted its antiproliferative effect by inhibiting the estrogen receptor α and HDAC2 mediated signaling cascade (Mam et al., 2020). Zhang et al. found crocetin (50 μmol/L) increased the suppressive effects on fluorouracil-treated MCF-7 cells, possibly through decreasing Beclin-1 levels increasing ATG1 levels (Zhang and Li 2017). In addition, crocetin (1, 10 μmol) significantly inhibited proliferation and invasion through downregulation of MMPs expression in MDA-MB-231 cells (Chryssanthi et al., 2010).

#### 5.2.2 Gastrointestinal Cancers

##### 5.2.2.1 Esophageal Cancer

Li et al. examined the anticancer effect of crocetin on esophageal squamous cell cancer cells (KYSE-150). After incubating KYSE-150 with crocetin (0, 12.5, 25, 50, 100, and 200 μmol/L) for 48 h, cell proliferation was decreased in a concentration-dependent manner, which was related to S-phase cell arrest. The expression of pro-apoptotic Bax was increased, and caspase-3 was activated, inducing apoptosis and cell morphology changes (Li et al., 2015). Further experiments were conducted to investigate the effect of combined crocetin and cisplatin on KYSE-150 cells. The combination of crocetin (200 μmol/L) and cisplatin (2 μmol/L) significantly reduced cell proliferation and induced apoptosis. Crocetin combined with cisplatin disrupted mitochondrial membrane potential, upregulated cleaved caspase-3 expression, and downregulated Bcl-2 expression. Moreover, the expression levels of p53 and p21 in combination therapy-treated KYSE-150 cells were significantly higher than those in cells treated with crocetin/cisplatin alone. In summary, the combination of crocetin and cisplatin exerted a synergistic anticancer effect by upregulating the p53/p21 pathway (Li et al., 2017).

##### 5.2.2.2 Gastric Cancer

Crocetin (50–240 μmol/L) inhibited the proliferation of gastric adenocarcinoma cells (AGS), increased the number of early apoptotic cells, and decreased the Bcl-2/Bax ratio in AGS cells. Following the treatment of chemically-induced rats with crocetin, the experimental results revealed that crocetin reversed changes in serum antioxidant activity and LDH levels in rats (Bathaie et al., 2013a). On treating SGC7901 cells with crocetin (12.5, 25, and 50 μmol/L) for 48 h, cell growth was markedly inhibited in the crocetin group in a concentration-dependent manner, which showed that the cell density decreased and the cell morphology became smaller and shrunk. In addition, the apoptosis rates of SGC7901 cells in the low-, medium-, and high-dose crocetin groups were 21.41, 28.28, and 39.83%, respectively. The apoptotic effect could be related to the activation of caspase-3, upregulation of Bax protein expression, and downregulation of Bcl-2 protein expression, thus reducing mitochondrial membrane potential and inducing cell apoptosis to produce anticancer effects (Zhang 2020). Zang et al. found that crocetin inhibited the proliferation, migration and invasion of gastric cells. Western blot analysis revealed that crocetin inhibited Sonic hedgehog (SHH) signaling with decreased SHH, PTCH2, Sufu, and Gli1 protein levels (Zang et al., 2021). In addition, studies have shown that crocetin has an apoptotic effect on BGC-823, indicating that crocetin can be used as an effective drug for treating gastric cancer (He et al., 2014).

##### 5.2.2.3 Colon Cancer

In a study by Zhuang et al., colon cancer cells (HCT116) were treated with 30 µM crocetin; the results showed that the cell proliferation rate decreased to 14% after 24 h, while fluorescence microscopy revealed that crocetin could induce the cell apoptosis. This phenomenon might be attributed to crocetin-mediated downregulation of inflammation-related genes. In addition, the expression levels of inflammation-related genes, HMGB1, IL-6, and IL-8, were significantly reduced following crocetin treatment of HCT-116 cells (Zhuang et al., 2018). Shao et al. used 1,2-dimethylhydrazine (DMH) to induce colorectal cancer in rats and showed that crocetin (5,10,20 mg/kg) treatment regulated the activity of antioxidant parameters, including SOD, GSH-Px, CYT-B5, CYP P450, glutathione-S-transferase (GST), and UDP-glucuronyltransferase (UDP-GT). The results showed that crocetin reduced the levels of COX-2, prostaglandin D_2_ (PGD-2), and NO. In addition, crocetin decreased the expression of apoptosis markers (caspase-3 and caspase-9) (Shao et al., 2021). Li et al., 2020 revealed that crocetin (0.8 mmol/L) inhibited the proliferation of SW480 cells by inducing S-phase arrest. One possible anti-tumor mechanism was that crocetin activated p21 in a P53- independent manner. Crocetin induced cytotoxicity in SW480 cells by promoting apoptosis and reducing the DNA repair ability (Li et al., 2012). Approximately 50% of mutation hotspots in colon cancer are located in p53 (Ekremoglu and Koc 2021). Based on a study by Ray et al., p53 caused Bax translocation and upregulated p53-induced death domain protein in p53 expressing cancer cells, subsequently resulting in cleavage and activation of t-BID through caspase-2. BAX and t-BID altered mitochondrial transmembrane potential, leading to caspase-9- and caspase-3 mediated apoptosis. However, in P53 damaged cancer cells, crocetin utilized P73 (P53 paralog) *via* the FAS-associated death domain to induce apoptosis in colon cancer (Ray et al., 2016).

##### 5.2.2.4 Pancreatic Cancer

Rangarajan et al. demonstrated that crocetin (10 μmol/L) reduced the size and number of nuclear globules in cancer stem cells (CSCs) and inhibited the expression of the marker protein DCLK-1, suggesting a targeting effect against CSCs. The mechanism of CSC inhibition might involve the binding of Sonic hedgehog (SHH) to cognate receptors, allowing the accumulation and activation of Gli transcription factors, which inhibited and smoothened SHH expression (Rangarajan et al., 2015). In an *in vitro* experiment, MIA-PaCa-2 cells were treated with crocetin for 72 h. The inhibition rates of crocetin on cell proliferation were 43, 59, and 71% at concentrations of 50, 100, and 200 μmol/L, respectively. After crocetin treatment, the distribution of S-phase cells decreased, confirming damaged DNA replication. As a checkpoint protein that regulates the G2-M cell cycle phase, enhancement of Cdc-2 phosphorylation and the inhibition of cyclin B1 might be the main factors underlying crocetin-induced G2-M phase arrest (Dhar et al., 2009). To further investigate the effect of crocetin on MIA-PaCa-2cells, the cells were injected into the right hind leg of nude mice, which were orally administered crocetin (4 mg/kg) for 30 days after the presence of palpable tumors. Tumor growth in crocetin-treated animal models was significantly reduced when compared with that in the control group. In addition, the number of proliferating cell nuclear antigen (PCNA)-positive cells in the crocetin group was enhanced, and the expression and phosphorylation of epidermal growth factor receptor were significantly decreased. The increase in the Bax/Bcl-2 ratio further highlighted the effect of apoptosis (Dhar et al., 2009).

#### 5.2.3 Cervical Cancer

HeLa cells were treated with crocetin (240 μmol/L) for 48 h, and the number of viable cells was reduced due to inhibited cancer cell proliferation. Crocetin increased the number of HeLa cells in the sub-G_1_ phase, thus indicating that crocetin inhibited cancer cell proliferation by inducing cell cycle arrest at the G1 phase, which might be mediated *via* P53 and its downstream p21WAF1/Cip1 expression. However, in SKOV3 cells lacking the P53 gene, crocetin activated p21WAF1/Cip1 *via* a p53 independent mechanism. The LDH release assay revealed that crocetin also enhanced cancer cell apoptosis and led to cell death. Moreover, the combination of crocetin and vincristine synergistically induced cell death. Accordingly, crocetin is a potential chemical preventive and anticancer agent when combined with vincristine (Zhong et al.). Kim et al. further confirmed that crocetin reduced the protein expression of LDHA in HeLa cells (Kim et al., 2014). Chen et al. used a methylcholanthrene (MCA)-induced cervical cancer model in mice and HeLa cervical cancer cells to examine the anticancer activity of crocetin (Chen et al., 2015). Previous studies have shown that several pathological diseases, including cervical cancer, are characterized by the activation of inflammatory pathways (Peng et al., 2019). Crocetin supplementation attenuated the serum levels of IL-1β, TNF-α, polymorphonuclear granulocytes (PMN), and nitrates, which are known to be increased in cancer models (Chen et al., 2015). Other studies have reported the upregulation of COX-2 expression in various cancers (Zhang et al., 2018). Crocetin can dose-dependently reduce the production of COX-2 in HeLa cervical cancer cells (Chen et al., 2015).

#### 5.2.4 Lung Cancer

The levels of lipid peroxidation and marker enzymes [aryl hydrocarbon hydroxylase (AHH), adenosine deaminase (ADA), gamma-glutamyltranspeptidase (GGT), and LDH] were significantly increased in benzo (a) pyrene-induced lung cancer animal models, which returned to near-normal levels following crocetin treatment. Crocetin [20 mg/kg (i.p.)] also increased the activities of GSH metabolic enzymes and antioxidant enzymes, which are known to be reduced in lung cancer models. Crocetin ameliorated the pathological changes observed in cancer models (Magesh et al., 2006). Magesh et al. examined the ability of crocetin to inhibit tumor formation and growth in mice with lung cancer. The animal models were intraperitoneally administered crocetin (50 mg/kg) for 3 days per week. The experimental results showed that after 8 or 18 weeks of crocetin treatment, cell proliferation decreased by 45 or 68%, respectively, which might be due to the inhibition of polyamine synthesis and glycoprotein changes (Magesh et al., 2010). Crocetin disodium salt was used to evaluate the growth inhibitory effect on A549 cells, with an IC_50_ value of 114.0 ± 8.0 μmol. The mechanism of action is related to LDH inhibition (Granchi et al., 2017).

#### 5.2.5 Liver Cancer

Kim et al. investigated the cytotoxicity of crocin and crocetin on HepG2 cells (hepatocellular liver cell line). The authors revealed that crocin and crocetin reduced the survival rate of HepG2 cells in a dose-dependent manner (Kim et al., 2014). Parizadeh et al. found that saffron extract had a cytotoxic effect against HepG-2 and Hep-2 cell lines, which may be associated with the reduced NO concentration (Parizadeh et al., 2011). STAT3 is a critical oncogenic transcription factor. Recent studies have shown that crocetin exerts antiproliferative activity by inhibiting STAT3 signaling in hepatocellular carcinoma. In hepatocellular carcinoma cells, crocetin (50 μmol) inhibited proliferation and promoted apoptosis. Furthermore, crocetin downregulated STAT3 activation and nuclear accumulation and inhibited its DNA-binding activity. In addition, crocetin suppressed the activity of upstream kinases (Src, JAK1, and JAK2). Another study showed that crocetin treatment suppressed STAT3 regulated genes expression, such as Bcl-2, Bcl-xL, cyclin D1, survivin, VEGF, COX-2, and MMP-9 (Mohan et al., 2021).

#### 5.2.6 Prostate Cancer

Studies have shown that saffron and crocin inhibit the proliferation of prostate cancer cells by blocking cell cycle progression and exerting anticancer activity (D'Alessandro et al., 2013). Claudio et al. studied the effect of crocetin on the tumor growth of two invasive PCa cell lines in male nude mice. Crocetin (30 mg/kg) was orally administered to cancer mice for 5 days. Following treatment, crocetin directly interfered with topoisomerase II to induce DNA damage and apoptosis, reverse epithelial-mesenchymal transition (EMT), increase E-cadherin expression, and significantly decrease the expression of N-cadherin and β-catenin. In addition, crocetin inhibited the migration and invasion of PCa cells by downregulating the expression of metalloproteinase and urokinase (Claudio et al., 2014).

#### 5.2.7 Ovarian Cancer

Neyshaburinezhad et al. encapsulated crocetin in poly (lactic-co-glycolic acid) nanoparticles (PLGA-Crt NPs) to investigate its resistance to cisplatin-resistant human ovarian carcinoma cell line (A2780-RCIS). The results showed that PLGA-Crt NPs (25, 50, 100, 200 μmol) could reduce the gene expression and efflux function of multidrug resistance protein 2 (MRP2) transporters in cisplatin-resistant A2780-RCIS to inhibit cell resistance (Neyshaburinezhad et al., 2019).

#### 5.2.8 Leukemia

The effect of crocetin on the proliferation and differentiation of HL-60 cells has been examined, revealing that 2 μmol crocetin inhibited cell growth by 50%. Crocetin (5 μmol) induced the differentiation of HL-60 cells, and the differentiation rate was 50% (Tarantilis et al., 1994).

Recent studies have shown that crocetin can be used as a candidate drug against primary acute promyelocytic leukemia (APL). Moradzadeh et al. found that crocetin (100 μmol/L) inhibited the proliferation of primary APL, NB4, and HL60 cells, which might be related to the reduced expression of prosurvival genes (Akt and BCL2), multidrug resistance proteins (ABCB1 and ABCC1), and inhibition of tyrosine DNA phosphodiesterase 1 (TDP1). Meanwhile, the increased expression of CASP3, CASP9, and the Bax/BCL2 ratio indicated that crocetin could induce cell apoptosis (Maliheh et al., 2019). Wen et al. reported that crocetin (10, 20 μg/ml) exerted anti-inflammatory effects in LPS-induced RAW264.7 cells. Inhibiting the MEK1/JNK/NF-κB/iNOS pathway and activating the Nrf2/HO-1 pathway could produce anti-inflammatory effects. Consequently, crocetin can be used as a potential redox balance regulator to exert anti-inflammatory and chemopreventive effects (Wen et al., 2021).

#### 5.2.9 Skin Cancer

Wang et al. examined the inhibitory effect of crocetin on 12-O-tetradecanoylphorbol-13-acetate (TPA)-induced skin tumors in female CD-I mice. Local application of crocetin (0.2 or 1.0 μmol) twice weekly for 20 weeks showed a tumor inhibition rate of 69% in TPA-induced mice. Pretreatment of cancer mouse skin with crocetin inhibited the production of myeloperoxidase and hydrogen peroxide (Wang et al., 1995). Tyrosinase is a pivotal enzyme in melanin biosynthesis (Ando et al., 2007). Protein levels of tyrosinase were reduced following crocetin treatment. Simultaneously, intracellular ROS levels were decreased, and crocetin was non-cytotoxic. Collectively, crocetin inhibits melanin production in B16F10 cells (Hashemi S. et al., 2018). Chu et al. examined the effects of crocetin and its derivatives formed by crocetin acylation with piperidine on B16F10 cells. The authors showed that the inhibitory rates of crocetin and its derivatives were 20.60 and 72.06%, respectively, which benefited tumor inhibition, as well as prevented metastasis in melanoma (Chu et al., 2018).

### 5.3 Nervous System

Although the pathogenesis of nervous system disease remains unclear, the potential role of crocetin has been discussed in subsequent studies (Table 4).

**TABLE 4:** Effect of crocetin on nervous system diseases.

Pharmacologic action	The subjects	Doses	Mechanism of action	References
Memory-enhancing effect	Rats with chronic cerebral hypoperfusion	Crocetin (8 mg/kg)	Protective effect on the cerebral cortex and hippocampal neurons	Mohajeri et al. (2013)
Alzheimer’s disease (AD)	SH-SY5Y and PC12 cells		Inhibits the active forms of GSK3β and ERK 1/2 kinases and significantly reduces the total tau protein and tau protein phosphorylation	Chalatsa et al. (2019)
	7PA2 cell	Crocetin (10 μmol)	Modulates the expression of CTF-α and CTF-β	Wong et al. (2020)
	HT22 cell		Reduces oxidative stress	Yoshino et al. (2014)
	Mouse hippocampal HT22 cell	Crocetin (1 and 5 μmol)	Improves the reduction of cell activity and mitochondrial membrane potential	Kong et al. (2014)
	CD14^+^ monocytes from Patients with AD	*Trans*-crocetin (5 μmol)	Upregulates lysosomal protease cathepsin B to promote the degradation of Aβ42	Tiribuzi et al. (2016)
Parkinson’s disease	Parkinson’s disease mice	Saffron pigment composition	Increases the number of tyrosine hydroxylase-positive neurons and enhances the dopamine content	Yao et al. (2018)
	Parkinson rats	Crocetin (25, 50, 75 μg/kg)	Increases the activities of antioxidant enzymes	Ahmad et al. (2005)
Cerebral injury	Cerebral contusion rats	Crocetin (50 mg/kg)	Inhibits neuronal apoptosis and promotes angiogenesis	Bie et al. (2011)
	Focal cerebral ischemia rats		Increases the activity of glutathione peroxidase (GSH-Px) and reduce the expression of caspase-3 mRNA and NF - κB	Tan and Li (2012)
Improved sleep quality	Patients with mild insomnia	Crocetin (7.5 mg/kg)	Contributes to maintaining the sleep continuity	Umigai et al. (2018)
	21 adult men with mild sleep problems	Crocetin (7.5 mg/kg)	Reduces the frequency of wakening episodes	Kuratsune et al. (2010)
Neuropathic pain	Mice		Reduces tumor necrosis factor (TNF)-α and interleukin (IL)-β and increases the activity of Mn superoxide dismutase (MnSOD)	Wang et al. (2017a)
Depression	Chronic restraint stress rats	Crocetin (20, 40, 60 mg/kg)	Restores malondialdehyde, glutathione, and antioxidant enzymes to normal levels	Wang et al. (2017b)
	Chronic stress mice	Crocetin (20, 40, 80 mg/kg)	Influences MKP-1/ERK1/2/CREB pathways	Lin et al. (2020)

#### 5.3.1 Memory-Enhancing Effect

Mohajeri et al. studied the memory-enhancing effect of crocetin in rats exhibiting chronic cerebral hypoperfusion. Vascular dementia was established by permanent ligation of the bilateral carotid arteries. The authors revealed that intraperitoneal administration of crocetin (8 mg/kg) significantly shortened the escape latency time in the Morris water maze. Histopathological analysis showed that crocetin had a good ischemic protective effect on the cerebral cortex and hippocampal neurons. In conclusion, crocetin treatment effectively prevented hippocampal neuropathy and improved spatial learning and memory in rats with chronic cerebral hypoperfusion (Mohajeri et al., 2013).

#### 5.3.2 Alzheimer

In the study by Chalatsa et al., two Alzheimer’s disease (AD) neuronal culture models, SH-SY5Y and PC12, were used to examine the potential effects of crocetin. SH-SY5Y cell overexpressing amyloid precursor protein showed that *trans*-crocetin (0.1 μmol–1 mmol) could affect the amyloidogenic pathway. *Trans*-crocetin treatment reduced β-secretase (BACE1) and γ-secretase (PSEN1 and PSEN2) and induced the accumulation of amyloid-β precursor protein (AβPP). In PC12 cells expressing hyperphosphorylated tau, *trans*-crocetin (0.1 μmol–1 mmol) effectively inhibited the active forms of GSK3β and ERK 1/2 kinases and significantly reduced total tau protein and tau protein phosphorylation (Chalatsa et al., 2019). In a similar experiment, crocetin was encapsulated in γ-cyclodextrin to determine its effectiveness in treating AD. Crocetin (10 μmol) and inclusion complex (10 μmol) modulated the expression of carboxyterminal fragments (CTF)-α and CTF-β in AD cell model (7PA2 cells). By reducing the expression level of CTF-β in 7PA2 cells, the level of amyloid-β (Aβ) produced by γ-secretase on cleaving CTF-β was downregulated. Crocetin and crocetin-γ-cyclodextrin exhibited protective effects against H_2_O_2_-induced cell death. Crocetin-γ-cyclodextrin (1.25–100 μmol) had no toxic effect on normal neuroblastoma cells (N2a cells and SH-SY5Y cells) (Wong et al., 2020). Studies have shown that the neurotoxicity of Aβ can be partly attributed to oxidative stress (Boyd-Kimball et al., 2005). One study revealed that crocetin-induced inhibition of Aβ1-42-induced hippocampal HT22 cell death could be mediated *via* reduced ROS production. In conclusion, crocetin afforded a neuroprotective effect against Aβ1-42-induced hippocampal cell cytotoxicity by reducing oxidative stress (Yoshino et al., 2014). The results showed that crocetin inhibited the formation of Aβ fibers and disrupted the stability of preformed Aβ fibers. In addition, crocetin stabilizes Aβ oligomers and prevents their conversion to Aβ fibers (Ahn et al., 2011). Crocetin (1 and 5 μmol) ameliorated the decreased cell activity and mitochondrial membrane potential, as well as the increased ROS formation, in HT22 cells induced by Aβ1-42. In addition, preliminary treatment with crocetin (5 μmol) activated the phosphorylation of ERK-1/2 (Kong et al., 2014). Tiribuzi et al. isolated CD14^+^ monocytes from 22 patients with AD presenting moderate cognitive impairment and found that *trans*-crocetin (5 μmol) promoted the degradation of Aβ42 in AD monocytes by upregulating lysosomal protease cathepsin B (Tiribuzi et al., 2016). Further studies showed that crocetin promoted the elimination of Aβ by inducing autophagy *via* the STK11/LKB1-mediated AMPK pathway (Wani et al., 2021). Crocetin (10–40 μmol) also inhibited NF-κB activation and P53 expression in the hippocampus of AD transgenic mice, reduced Aβ secretion, and ameliorated memory and learning ability (Zhang et al., 2018).

In summary, crocetin seems to confer a beneficial effect on multiple therapeutic targets for AD. Therefore, this compound is promising for the treatment of AD.

#### 5.3.3 Parkinson’s Disease

Yao et al. reported that the saffron pigment composition extracted from plants could significantly improve dyskinesia, increase the number of tyrosine hydroxylase-positive neurons in the substantia nigra, and increase dopamine (DA) content in the striatum of mice. Therefore, saffron pigment composition has a therapeutic effect on 1-methyl-4-phenyl-1, 2, 3, 6-tetrahydropyridine-induced Parkinson’s disease (PD) mice (Yao et al., 2018). Abnormal aggregation of α-synuclein (αS) in the nervous tissue is known to result in neurodegenerative diseases, such as PD (Schulz-Schaeffer 2010). The effects of crocetin on αS polymerization and αS fibril dissociation were examined, revealing that crocetin inhibited the aggregation and dissociation of αS fibrils in a dose-dependent manner, as determined by thioflavin T fluorescence. Transmission electron microscopy showed that αS fibers were decreased and shortened (Inoue et al., 2018). Ahmad et al. used 6-hydroxydopamine (6-OHDA)-induced PD to examine the neuroprotective effect of crocetin. The crocetin [25, 50, and 75 μg/kg (i.p.)] treatment group exhibited significantly improved walking speed and distance in rats. The activities of antioxidant enzymes [GSH-Px, GSH reductase (GR), GST, CAT, and SOD] were increased in the striatum, and the levels of DA and its metabolites were effectively protected. In the substantia nigra, the content of thiobarbituric acid reactive substances was reduced. The histopathological results showed that crocetin protected neurons from 6-OHDA-induced injury (Ahmad et al., 2005). Dong et al. revealed that crocetin afforded potential therapeutic effect against 1-methyl-4-phenyl-1,2,3,6-tetrahydropyridine (MPTP)-induced PD by improving mitochondrial function. Crocetin [50, 100 mg/kg (p.o.)] reduced MPTP-induced motor deficits and protected dopaminergic neurons in PD model mice. The mRNA expression levels of IL-1 β, IL-6, IL-10, TNF-α, inducible NOS (iNOS), and COX-2 were increased; however, crocetin treatment reversed these changes in the *in vivo* and *in vitro* models. Furthermore, crocetin treatment regulated mitochondrial permeability transition pore activity in an ANT- and cyclophilin D-dependent manner to prevent mitochondrial dysfunction (Dong et al., 2020).

#### 5.3.4 Cerebral Injury

Bie et al. showed that crocetin [50 mg/kg (i.g.)] regulated the expression of Bcl-2 protein, suggesting the inhibition of neuronal apoptosis in rats exhibiting cerebral contusions. Crocetin increased the expression levels of serum response factor and VEGFR-2. Based on the experimental findings, the protective effect of crocetin on cerebral contusion may be associated with the inhibition of neuronal apoptosis and the promotion of angiogenesis (Bie et al., 2011). Tan et al. performed intragastric administration of crocetin once daily for 7 days. Induction of focal cerebral ischemia (for 2 h) and reperfusion (for 22 h) was established by occlusion of the middle cerebral artery with a thread embolism. Crocetin was found to reduce the cerebral infarction volume and improve neurological function, potentially protecting against cerebral ischemia-reperfusion injury by increasing GSH-Px activity and decreasing the expression of caspase-3 mRNA and NF-κB in brain tissues (Tan and Li 2012). Similar experiments have shown that crocetin reduced the MDA and NO content and enhanced the SOD activity in brain tissue (Tan et al., 2011).

#### 5.3.5 Sleep Quality Improvement

To study the effect of crocetin on sleep in patients with mild insomnia, Naofumi et al. conducted a randomized, double-blind, placebo-controlled study, randomly dividing 30 participants into two groups. Each group was prescribed crocetin or a placebo at 7.5 mg/day. The results of objective sleep parameters measured by single-channel encephalography (EEG) showed that crocetin enhanced delta activity, which contributed to the maintenance of sleep continuity. Using the Oguri-Shirakawa-Azumi sleep inventory MA version (OSA-MA) to evaluate subjective sleep parameters, crocetin could improve sleepiness and afford a refreshed feeling when participants woke up. Studies have shown that crocetin can help maintain sleep and improve sleep quality (Naofumi et al., 2018). Kuratsune et al. studied the influence of crocetin on sleep in 21 adult males with mild sleep problems. Participants were given crocetin capsules (crocetin content: 7.5 mg/kg) to complete a double-blind, placebo-controlled crossover trial for 6 weeks. The results showed that the frequency of wakening episodes was significantly lower in the crocetin group than in the placebo group. According to the subjective sleep questionnaire data, crocetin can ameliorate sleep quality without obvious side effects (Kuratsune et al., 2010).

#### 5.3.6 Neuropathic Pain

Wang et al. studied the effect of crocetin in a mouse model of spared nerve injury (SNI)-induced neuropathic pain. The authors revealed that crocetin reduced thermal tenderness and mechanical properties in SNI mice. Crocetin treatment reversed the increased TNF-α and IL-β levels induced by SNI. Crocetin also increased the activity of manganese SOD (MnSOD) in the mitochondria of the spinal cord and sciatic nerve in mouse models. In conclusion, crocetin could potentially attenuate neuropathic pain (Wang F. X. et al., 2017).

#### 5.3.7 Depression

Farkhondeh et al. examined the effect of crocetin on chronic restraint stress-induced depression in rats. The rats were placed in restrainers for 1 h each day for 21 days. The animals were injected with crocetin (20, 40, and 60 mg/kg) daily. Treatment with crocetin improved the immobility time in rats subjected to chronic stress and restored brain MDA, GSH, and antioxidant enzyme levels to normal when compared with the non-treated group. The antidepressant effect of crocetin is related to its antioxidant activity (Farkhonde et al., 2018). In addition, the relationship between crocetin and the gut microbiota-brain axis in mediating antidepression-like actions was established. Crocetin (20, 40, and 80 mg/kg) improved the depressive behavior in mice subjected to chronic restraint stress depression, and histopathological analysis showed that crocetin afforded a protective effect on hippocampal neuronal cells. The expression of ERK 1/2 and cAMP-response element binding protein (CREB) was elevated, while the hippocampal expression of MAPK phosphatase 1 (MKP-1) and pro–brain-derived neurotrophic factor (proBDNF) was suppressed. Numerous studies have shown that intestinal ecosystem disorders strongly correlate with depression (Herman 2019). Crocetin can increase the abundance of Bacteroidetes, Enterobacteriaceae, and Saccharimonadaceae in depressed mice and secrete neurotransmitters related to depression, such as GABA, serotonin, and norepinephrine. These findings indicate that crocetin influences intestinal microflora metabolism and composition, and the regulation of intestinal microbiota refers to the expression of proteins related to the MKP-1/ERK1/2/CREB pathway (Lin et al., 2020).

### 5.4 Ocular Pathologies

#### 5.4.1 Myopia Prevention

In a study by Mori et al., 69 participants, aged 6–12 years, were randomized to receive either placebo or crocetin and followed for 24 weeks in a multicenter, double-blind, placebo-controlled clinical trial (Table 5). The results showed that spherical equivalent refraction (SER) was smaller in the crocetin group (−0.41 ± 0.05 diopter) than in the placebo group (−0.33 ± 0.05 diopter). The axial length (AL) elongation was significantly smaller in the crocetin group (0.18 ± 0.02 mm) than that in the placebo group (0.21 ± 0.02 mm). In conclusion, dietary crocetin may have a therapeutic effect on myopia in children (Mori et al., 2019).

**TABLE 5:** Effect of crocetin on ocular pathologies diseases.

Pharmacologic action	The subjects	Doses	Mechanism of action	References
Myopia prevention	69 participants aged 6–12 years		Changes spherical equivalent refractions (SER) and axial length (AL)	Mori et al. (2019)
Proliferative vitreous retina	ARPE-19cells		Inhibits the activation of p38MAPK to antagonize the epithelial-mesenchymal transition	Wang (2018)
	Rabbit PVR models	Crocetin (0.2 and 0.4 μmol)		Wang (2018)
Age-related macular disease	RGC-5 cells	Crocetin (3 μmol)	Inhibits the damage to RGC-5 cells and suppresses the increase in caspase-3 and caspase-9 activities	Yamauchi et al. (2011)
	Retinal injury mice	Crocetin (100 mg/kg, p.o.)	Reduces the number of TUNEL-positive cells and inhibits retinal dysfunction and photoreceptor degeneration	Yamauchi et al. (2011)
Retinal damage	Retinal injury mice	Crocetin (20 mg/kg, p.o.)	Improves the decrease in the number of ganglion cell layer cells and thickness of the inner nuclear layer	Zhang et al. (2018)
	Retinal injury mice	Crocetin (20 mg/kg, p.o.)	Reduces the phosphorylation of MAPK, JNK, and p38	Ishizuka et al. (2013)
Retinal edema	the RVO mouse model	crocetin (100 mg/kg)	Decreases the expression of matrix metalloproteinase (MMP-9) and tumor necrosis factor (TNF-α) increase the expression of occludin	Nitta et al. (2019)
Glaucoma	retinal injury models	Crocetin (100 mg/kg)	Inhibits caspase-3/7 and the expression of cleaved caspase-3	Ohno et al. (2012)
	OHT mouse model	Saffron extract	Prevents the downregulation of P2RY12 expression and retinal ganglion cell death	Fernández-Albarral et al. (2019)
Diabetic retinopathy	Diabetic rats	Crocetin (50,100 mg/kg)	Inhibits the expression of TNF-α, Bax, and caspase-3 and increases the expression of Bcl-2	Zhao et al. (2020b)

#### 5.4.2 Proliferative Vitreous Retina

Wang et al. studied the inhibitory effect and molecular mechanism of crocetin on proliferative vitreoretinopathy in ARPE cells and rabbit proliferative vitreous retina (PVR) models. *In vitro*, crocetin inhibited the proliferation of ARPE-19 cells by blocking the cell cycle in the G1 phase, upregulating the expression of p53 and its downstream p21, and inhibiting PCNA expression. In addition, crocetin inhibited the horizontal and vertical migration of ARPE-19 cells. Crocetin inhibited the activation of p38MAPK to antagonize the EMT induced by transforming growth factor (TGF)-β2 in ARPE-19 cells. *In vivo*, the results revealed that the intravitreal injection of 0.2 and 0.4 μmol crocetin did not damage the structure and function of the rabbit retina. Special ophthalmic examinations were performed on days 7 and 14 after injection. Optical coherence tomography revealed no vitreous opacity, clear structure of retinal layers, edema and optic atrophy, and retinal hemorrhage. Histopathological results showed that the structure of retinal layers in experimental eyes and control eyes were intact, along with the absence of thinning of inner and outer nuclear layers, retinal atrophy, and inflammatory cell infiltration (Wang 2018).

#### 5.4.3 Age-Related Macular Disease

Age-related macular degeneration (AMD) is the main cause of visual impairment in the elderly. Yamauchi et al. investigated the effect of crocetin on RGC-5 cell death induced by tunicamycin, H_2_O_2_, and light-induced retinal injury in mice, *in vivo* and *in vitro*. Crocetin (3 μmol) significantly inhibited the damage of RGC-5 cells and suppressed the increase in caspase-3 and -9 activities. *In vivo*, white light at 8000 lx was used to induce retinal damage. Crocetin [100 mg/kg, peroral (p.o.)] significantly reduced the number of TUNEL-positive cells and inhibited retinal dysfunction and photoreceptor degeneration. Crocetin has a potential therapeutic effect on AMD and other retinal degenerative diseases (Yamauchi et al., 2011). Crocetin pretreatment protected ARPE19 cells from t-butyl hydroperoxide (TBHP)-induced oxidative stress through intracellular ATP depletion, LDH release, cytoskeleton loss, and nuclear condensation. The underlying mechanism of action potentially involved protecting the cellular energy production pathway and activating the ERK1/2 pathway (Karimi et al., 2020).

#### 5.4.4 Retinal Damage

Crocetin (20 mg/kg, p.o.) improved the reduced number of ganglion cells and the thickness of the inner nuclear layer following I/R-induced retinal injury in mice. The electroretinogram (ERG) results showed that crocetin could prevent the decrease in A and B wave amplitudes. In addition, crocetin reduced the phosphorylation levels of p38, JNK, NF-κB, and c-Jun in the I/R-injured retina. These results suggest that crocetin prevents I/R retinal injury by inhibiting oxidative stress (Zhang et al., 2018). Likewise, oral administration of 20 mg/kg crocetin exhibited an inhibitory effect on I/R-induced retinal cell death and reduced the phosphorylation of MAPK, JNK, and p38 (Ishizuka et al., 2013).

#### 5.4.5 Retinal Edema

Nitta et al. found that oral administration of 100 mg/kg crocetin decreased the expression of MMP-9 and TNF-α and increased the expression of occludin in the retinal vein occlusion (RVO) model in mice. The results indicated that crocetin improved retinal edema and protected retinal tight junctions in RVO mice by inducing an anti-inflammatory effect (Nitta et al., 2019).

#### 5.4.6 Glaucoma

Selective retinal ganglion cells (RGCs) are a common feature of glaucoma. Previous studies have shown that intravitreal injection of N-methyl-D-aspartic acid (NMDA) can cause RGC loss (Lam et al., 1999). Accordingly, NMDA-induced retinal injury models were employed to determine the potential effect of crocetin on glaucoma. Histological analysis showed that crocetin (100 mg/kg) inhibited the NMDA injection-induced decrease in ganglion cell layer (GCL) cells. In addition, the number of TUNEL-positive cells was increased in the GCL and inner nuclear layer following NMDA injection; this effect was inhibited by crocetin. NMDA injection excited caspase-3/7 and enhanced the expression of cleaved caspase-3 in GCL cells; these processes were reversed by crocetin. In conclusion, orally administered crocetin prevented NMDA-induced retinal injury by inhibiting the caspase pathway, thereby inhibiting apoptosis of the GCL (Ohno et al., 2012). In addition, it has been shown that microglial activation in the retina might lead to RGC death. Albarral et al. studied the effect of a hydrophilic saffron extract containing 3% crocin on unilateral laser-induced ocular hypertension (OHT) mouse models. Saffron extract prevented the downregulation of P2RY12 expression and retinal ganglion cell death in OHT-induced eyes by reducing neuroinflammation associated with elevated intraocular pressure (Fernández-Albarral et al., 2019). Himori et al. showed that oral administration of antioxidant supplements (hesperidin, crocetin, and Tamarindus indica) for 8 weeks was effective in 30 patients with glaucoma exhibiting high oxidative stress levels. Dietary supplementation may be a promising strategy for treating oxidative stress-related diseases (Himori et al., 2021).

#### 5.4.7 Diabetic Retinopathy

To establish a diabetic retinopathy model, Sepahi et al. used RPE cells exposed to high glucose levels. As a result, VEGF gene expression and protein levels were reduced in the crocin and crocetin treatment groups. In addition, crocetin and crocin reduced the levels of MMP-2 and MMP-9, known factors of inflammation and angiogenesis (Sepahi et al., 2021). In the study by Zhao et al., intragastric crocetin (50, 100 mg/kg) was administered to streptozotocin (STZ)-induced diabetic rat models for 8 weeks. In the crocetin treatment group, the expression of TNF-α, caspase-3, protein kinase C (PKC), and Bax was significantly decreased, while the expression of Bcl-2 was increased in the retinal neuroepithelium (Zhao Y. J. et al., 2020).

### 5.5 Liver Protection

Crocetin displayed protective effects against aflatoxin B1-induced hepatotoxicity in rats by elevating the cytosolic GSH, as well as GST and GSH-Px activities (Wang et al., 1991). Sreekanth et al. examined the protective effect of crocetin on dengue virus (DENV)-infected liver damage in mouse models. Crocetin (50 mg/kg) was found to balance antioxidant enzymes (SOD and CAT), reduce the expression of pro-inflammatory cytokines, and inhibit nuclear translocation of NF-κB. The results showed that crocetin treatment could not reduce DENV replication in the liver of DENV-infected mice; however, crocetin could improve liver injury by reducing hepatocyte apoptosis (Sreekanth et al., 2020). In a study by Gao et al., the hepatoprotective effect of crocetin on paraquat (PQ) poisoned rats was investigated. The authors revealed that 50 mg/kg crocetin exerted hepatoprotective effects in PQ-poisoned rats, which may be achieved by reducing the levels of inflammatory factors in the blood and inhibiting the activities of caspase-8, -9, and -12, as well as the expression of iNOS and NF-κB in liver tissues (Gao et al., 2016). Liu et al. evaluated the protective effect of crocetin on arsenic trioxide (ATO)-induced hepatic injury and showed that 50 mg/kg crocetin could alleviate weight loss and hepatic pathological injury in rats with hepatic injury. Crocetin reversed the increase in alanine aminotransferase (ALT), aspartate aminotransferase (AST), and alkaline phosphatase. In addition, crocetin enhanced antioxidant and anti-inflammatory effects in the body by activating the Nrf2 signaling pathway (Liu P. et al., 2020). Guo et al. found that crocetin promoted autophagy in injured hepatocytes and reduced further hepatocyte damage (Guo et al., 2018). In addition, crocetin impacted non-alcoholic fatty liver cells and showed that the TG content in fatty liver cells was decreased, and lipid deposition was effectively alleviated; the underlying mechanism might be related to the reduction in cellular oxidative stress (Liao et al., 2011). Gao et al. found that crocetin can be used as a preventive drug for fulminant hepatic failure (FHF). The authors revealed that crocetin pretreatment improved the liver tissue morphology, decreased total bilirubin production, and reduced the activities of ALT and AST in FHF rats. Moreover, crocetin reduced hepatocyte apoptosis, p53 mRNA expression, and caspase family protein expression. In addition, crocetin decreased the secretion of inflammatory cytokines by inhibiting NF-κB activation and suppressing liver oxidative stress (Gao et al., 2019). Crocetin effectively alleviated the degree of liver injury and fibrosis in liver fibrosis mice, which might be related to the downregulation of p38MAPK protein expression (Wang X. et al., 2017).

### 5.6 Kidney Protection

Michael et al. reported that I/R-induced renal damage was reduced following treatment with 50 mg/kg crocetin. The results showed that crocetin could suppress inflammatory components and the degree of epithelial injury, as well as induce the expression of miR21, miR127, and miR132 (Michael et al., 2020). Wang et al. administered 50 mg/kg crocetin through the duodenum to rats with hemorrhagic shock and resuscitation. Crocetin improved renal dysfunction caused by hemorrhagic shock and resuscitation by restoring T-SOD activity and quenching the superoxide anion/free radical, inhibiting NF-κB activation, and preventing TNF-α and IL-6 production (Wang et al., 2012). Liu et al. found that crocetin could prevent ATO-induced renal injury by inhibiting oxidative stress, inflammation, and apoptosis, which may be associated with activation of the PI3K/Akt signaling pathway (Liu Y. et al., 2020).

### 5.7 Diabetes

Accumulated evidence has revealed that saffron and its extracts are beneficial for treating diabetes and its complications (Hashemi and Hosseinzadeh 2019; Kumar and Gupta 2019). The underlying mechanisms may involve stimulating glucose uptake by peripheral tissues, inhibiting endogenous glucose production, reducing insulin resistance, and stimulating islet β cells to release more insulin (Farkhondeh and Samarghandian 2014).

Elgazar et al. found that aqueous saffron extract significantly increased body weight and serum insulin levels, decreased blood glucose levels, improved lipid levels, as well as liver and kidney functions in alloxan-induced diabetic rats (Elgazar et al., 2013). Xi et al. reported that crocetin has a regulatory effect on high-fructose diet-induced insulin resistance and free fatty acid-induced insulin insensitivity. Crocetin restored the levels of adiponectin (an insulin-sensitizing adipocytokine), TNF-α, and leptin in the experimental group (Xi et al., 2007). In addition, Sheng et al. showed that crocetin accelerated the uptake and oxidation of TGs and non-esterified fatty acids in the liver, thereby increasing insulin sensitivity (Sheng et al., 2008). In addition, crocetin suppressed the palmitate-induced activation of c-Jun NH (2)-terminal kinase (JNK) and inhibitor kappaB kinase beta (IKKbeta) by inhibiting protein kinase Ctheta (PKCtheta) phosphorylation and improving insulin sensitivity in 3T3-L1 adipocytes (Yang et al., 2010).

Endothelial progenitor cell (EPC) dysfunction is an important risk factor for diabetic vascular complications; thus, Cao et al. investigated the role of crocetin in diabetic EPC dysfunction. EPCs were isolated from the bone marrow of diabetic mice. Crocetin (5 μM) treatment alleviated diabetic EPC proliferative damage. Furthermore, crocetin augmented LDH release, cell apoptosis, and caspase-3 activity. The mechanism of crocetin against the impairment in diabetic EPCs could involve enhanced NO bioavailability by regulating the PI3K/AKT-eNOS and ROS pathways (Cao et al., 2017). Similarly, crocetin (0.1, 1.0 μM) prevented high glucose-induced apoptosis of HUVECs, possibly associated with p-Akt activation, following upregulated eNOS and NO production (Meng and Cui 2008).

Zheng et al. investigated the therapeutic effect of crocetin on STZ-induced gestational diabetes mellitus (GDM) in rats. Crocetin reduced blood glucose levels and increased body weight in GDM rats. In addition, crocetin treatment increased the levels of antioxidant enzymes, including SOD, GSH-Px, GSH, and CAT, decreased expression levels of IL-6, TNF-α, and IL-1β, and suppressed the levels of intercellular adhesion molecule-1 (ICAM-1), COX-2, and PGE_2_. In addition, crocetin treatment enhanced levels of Bcl-2 and reduced levels of Bax and caspase-3 in rats. In summary, crocetin showed significant therapeutic effects against GDM by improving the status of endogenous antioxidant enzymes, inhibiting the inflammatory reaction, and suppressing mitochondrial pathway apoptosis (Zheng et al., 2021).

Mahdavifard et al. found that MB-92 (a combination of some amino acids and crocetin) has potential therapeutic effects for inhibiting glycation and oxidation products, atheromatous plaque formation, and inflammation in diabetic atherosclerotic rats (Mahdavifard et al., 2016).

Previous studies have shown that advanced glycation end-products (AGEs) are key pathogenic factors in diabetic angiopathy. Crocetin can inhibit the migration of AGE-induced VSMCs by suppressing receptor advanced glycation end (RAGE) expression, resulting in the reduction of protein levels of TNF-α and IL-6, as well as the suppression of MMP-2/9 activity (Xiang et al., 2017). Xiang et al. investigated the effect of crocetin on AGE formation and the expression of RAGE protein in diabetic rats. STZ-induced diabetic rats were intragastrically administered crocetin (50 mg/kg) for 21 days. Crocetin markedly reduced the content of fructosamine (FMN) and glycosylated hemoglobin (GHb), intermediate AGE products. In addition, the deposition of AGEs in the aortic and mesenteric vascular beds decreased, while the expression of RAGE was significantly decreased. Therefore, crocetin could afford a protective effect on blood vessels of diabetic rats (Xiang et al., 2006).

### 5.8 Other Applications

Mesenchymal stem cells (MSCs) play an important role in bone repair. Studies have reported that crocetin can effectively promote osteogenic differentiation of MSCs (Kalalinia et al., 2018). For example, Li et al. induced arthritis by administering intraperitoneal Complete Freund’s adjuvant in rats. The authors showed that crocetin could adjust paw edema and body weight in rat models in a dose-dependent manner. Crocetin protected rat models of arthritis by reducing HO-1/Nrf-2 expression and inhibiting inflammatory mediators (Li et al., 2018). Regulatory T cells (Tregs) are key regulatory factors in asthma. Ding et al. used crocetin to treat ovalbumin (OVA)-induced asthma in mice. Crocetin alleviated the asthma severity in mice. A possible mechanism underlying this effect is that crocetin activates Foxp3 through TIPE2 in Treg cells (Ding et al., 2015). In addition, crocetin has a potential therapeutic effect on scleroderma; crocetin (0.1, 1, or 10 μmol) inhibited the proliferation and differentiation of skin fibroblasts isolated from patients with systemic scleroderma in a concentration-dependent manner. Intraperitoneal injection of 50 mg/kg crocetin reduced skin and lung fibrosis in bleomycin-induced scleroderma mice, mainly owing to the reduction of endothelin-1 (ET-1) (Song et al., 2013). Crocetin has a protective effect against 2,4,6-trinitrobenzene sulfonic acid-induced colitis in mice. Studies have shown that 50 mg/kg crocetin (i.g.) significantly improved diarrhea and destruction of colon structure, as well as reduced the degree of neutrophil infiltration and lipid peroxidation in the inflammatory colon, thus suggesting that crocetin plays beneficial roles in experimental colitis (Kazi and Qian 2009). Previous findings have shown that saffron (*C. sativus* L.) extract has antinociceptive effects. Erfanparast et al. showed that crocetin injection into the cerebral fourth ventricle improved formalin-induced orofacial pain in rats, and the antinociceptive effect was related to central H_2_ histaminergic and α_2_ adrenergic receptors (Erfanparast et al., 2020).

## 6 Pharmacokinetics

**FIGURE 4:**
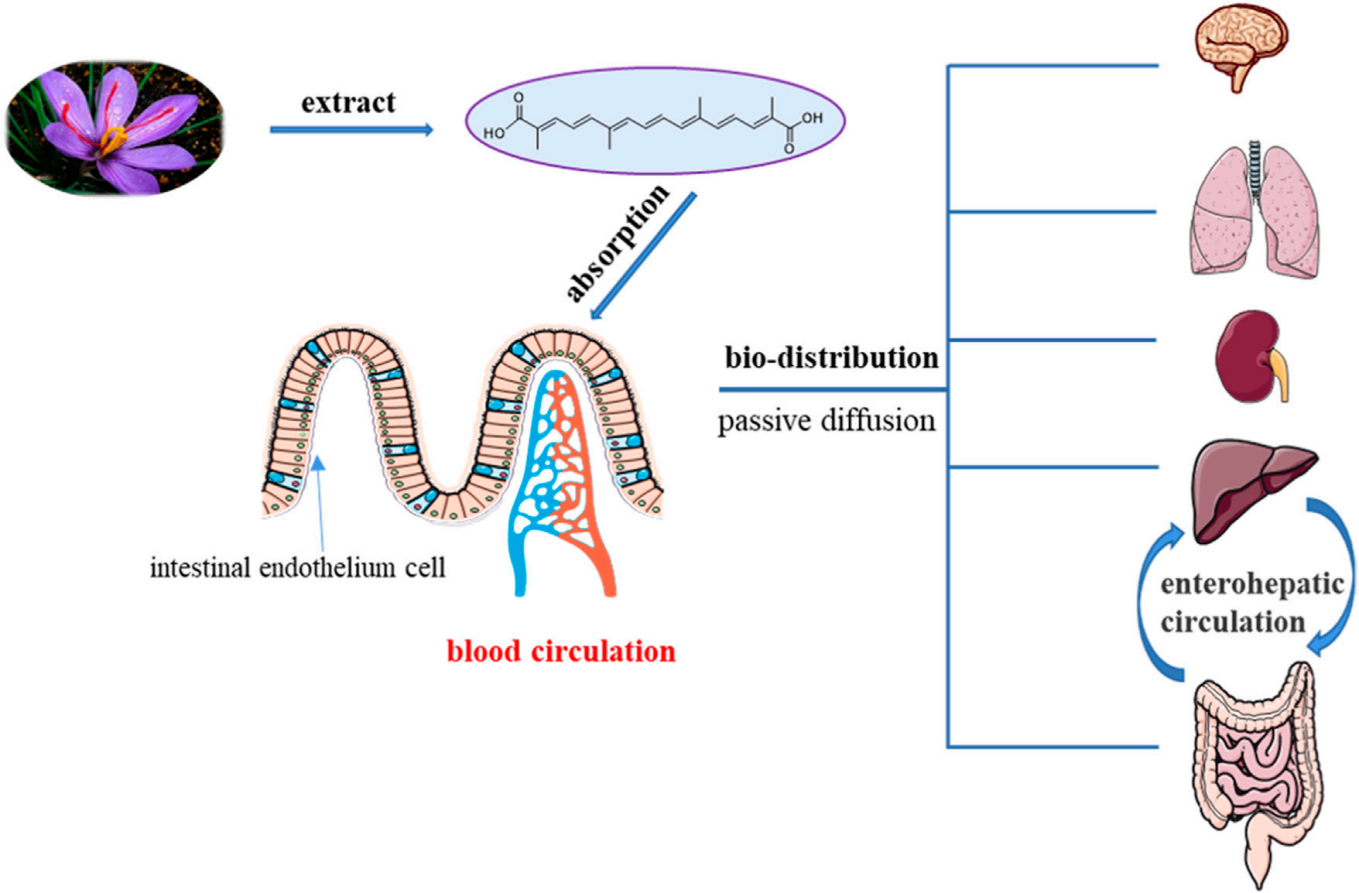
Pharmacokinetics of crocetin in the body.

To date, few experimental studies have assessed the pharmacokinetics of crocetin, a low molecular mass carotenoid (Almodóvar et al., 2020). Accumulated pharmacokinetic and pharmacological activity reports have shown that crocetin, the glycogen of crocin, is a bioactive metabolite of crocin that can exert therapeutic benefits (Razavi and Hosseinzadeh 2015; Yue et al., 2016) (Figure 4).

### 6.1 Absorption

1) Animal/cell research

In a report by Zhang et al., following intragastric administration of 25 mg/kg crocetin, Sprague Dawley (SD) rats reached the highest blood concentration (3.56 μg/ml) after 1.7 h; however, its oral bioavailability was only 11.25%. The area under the concentration-time curve from time zero to the last measurable concentration (AUC_0-t_) was 92.242 μg/L·h, AUC_0-∞_ was 92.244 μg/L·h (Zhang 2017).

Liu et al. administered crocetin (50 mg/kg) to 10 rats *via* intragastric administration. The content of crocetin in the plasma was determined using HPLC. The pharmacokinetic parameters were obtained by calculation; the half-life was approximately 30 min, the peak time was approximately 65 min, the maximum plasma concentration was 50 μg/ml, AUC_0-t_ was 845 ± 109 μg·min·ml^−1^, and volume of distribution (V_D_) was 50 ± 08 L kg^−1^ (Liu and Qian 2003).

To evaluate the effect of crocetin on cerebral I/R injury, six rats in each group were intravenously administered crocetin (0.33 mg/kg), and the total urine and feces samples were collected every 8 h after administration. Crocetin was not excreted in the urine or feces following intravenous administration. Moreover, it did not exhibit any anticipated pharmacological effects. Therefore, oral administration of crocetin is superior to intravenous administration (Zhang et al., 2019a).

Oliveira et al. determined the gastrointestinal absorption of major carotenoids (crocetin, crocin-1, and crocin-2) in *G. jasminoides* by assaying the transport using MKN-28 and Caco-2 cells lines. In general, crocetin showed the greatest efficiency in terms of gastrointestinal transport (Oliveira et al., 2017).

Lautenschläger et al. examined the intestinal permeation of *trans*-crocetin using a Caco-2 monolayer cell culture. The results showed that *trans*-crocetin permeated the intestinal barrier by transcellular passage, with approximately 32% of the substrate transported within 2 h. In addition, porcine brain capillary endothelial cells (BCECs) and the blood-cerebrospinal fluid barrier (BCSFB) were used to study the permeation characteristics of *trans*-crocetin across the blood-brain barrier (BBB). *Trans*-crocetin permeates the BBB to enter the central nervous system (CNS) at a slow but constant velocity over a 29-h period (Lautenschläger et al., 2015).

2) Clinical research

In a study by Almodóvar et al., 13 healthy human volunteers were administered different concentrations of saffron extract (56 and 84 mg), and blood samples were collected every 30 min after the first 3 h madministration. Crocin, safranal, and picrocrocin levels were undetectable in mplasma. Only sufficient concentrations of crocetin could be detected in blood samples to be identified and quantified. HPLC- photodiode-array detection and electrospray (PAD)/mass spectroscopy (MS) was used for identification and quantification. Approximately 60–90 min after oral administration, the maximum concentration (C_max_) of crocetin in blood could be detected, and the kinetics of the reaction was dose-dependent. According to the two doses, the mean C_max_ and the estimates of the pharmacokinetic parameters (AUC_0-3h_) of crocetin approximately ranged between 0.26 and 0.39 μg/ml and 21.07–26.15 μg·h/ml, respectively (Almodóvar et al., 2020).

The C_max_ of crocetin was 0.28 μg/ml with a single oral dose of 22.5 mg (Umigai et al., 2011). These data closely correlated with the C_max_ detected by Almodóvar et al. using saffron extract containing only 23 mg crocin, thus confirming that crocetin derived from saffron-extracted crocin was more bioavailable than the pure crocetin following oral administration. This finding could be explained by the greater bioavailability of crocin into enterocytes for later absorption than that of crocetin (Almodóvar et al., 2020).

The value of T_max_ after crocetin administration was smaller than that of other carotenoids, indicating that the absorption and detection of crocetin in plasma were more rapid than that of other carotenoids (Umigai et al., 2011).

### 6.2 Bio-Distribution

Miller et al. used absorption and fluorescence techniques to study the binding of crocetin to human and bovine plasma albumin. The results showed that crocetin binds to plasma albumin by occupying the binding site of free fatty acid binding, indicating that plasma albumin may be a transporter of crocetin (Miller et al., 1982). Hydrophobic interactions are one mechanism of crocetin and plasma albumin interaction (Kanakis et al., 2007).

Once in circulation, given the weak interaction between crocetin and plasma albumin, crocetin can reach different tissues and cross the BBB in a concentration-independent manner by passive transcellular diffusion mechanism, as demonstrated in an *in vitro* study. In addition, it should be noted that the typical transporter saturation effect could not be determined owing to the poor solubility of crocetin (Lautenschläger et al., 2015). However, similar studies have shown that crocetin is easily absorbed by intestinal epithelial cells, and its uptake is positively correlated with increased drug concentration, demonstrating that crocetin enters cells through passive diffusion (Wang HF. et al., 2018).

To determine whether absorption and transport of crocetin in the Caco-2 cell model is related to P-glycoprotein (P-gp), Lautenschläger et al. suggested that crocetin serves as the substrate of the PGP efflux pump and enters the BBB *via* passive transcellular diffusion (Lautenschläger et al., 2015). However, in another experiment, the apparent permeability coefficient of crocetin in the Caco-2 cell model was 5.06 × 10^−6^ cm·sec^−1^ and permeability damage rate (PDR) was 1.52, which indicated that crocetin was moderately absorbed in the Caco-2 cell model. After the addition of verapamil (P-gp inhibitor), the values of PappAP-BL and PDR did not significantly differ from those previously reported, which indicated that crocetin uptake and transport were not mediated *via* P-gp (Wang S. L. et al., 2018). This result contradicts the previous research by Lautenschläger. Accordingly, whether P-gp transporters or other intestinal transporters, such as MRPs and PEPT1, mediate crocetin absorption in the intestine warrants further study.

Christodoulou et al. examined the oral and intravenous administration of saffron (*C. sativus* L.) aqueous extract in C57/BL6J mice by assessing the kinetics of crocetin and its metabolites, exhibiting a one-compartment pharmacokinetic model with first-order absorption after oral administration. After intravenous administration of the aqueous saffron extract, the one-compartment pharmacokinetic model described the kinetics of crocetin, while the first-order kinetic parameters described the rate of crocetin to that of its metabolite (Christodoulou et al., 2019).

In a study by Zhang et al., cerebral I/R injury rats were administered crocin orally or intravenously, and neither crocin nor crocetin was detected in the cerebral tissue, indicating that crocin does not affect the cerebral tissue through its prototype or metabolite. More importantly, the effects of crocetin in the circulatory system might mediate the cerebral-protective effects (Zhang X. et al., 2019). However, Lautenschläger et al. demonstrated that *trans*-crocetin could cross the BBB to reach the CNS. The authors employed porcine BCECs and BCSFB as suitable models for monitoring the permeation characteristics of *trans*-crocetin across the BBB. The results showed that *trans*-crocetin bypassed the BBB at a slow but constant speed within 29 h (Lautenschläger et al., 2015). Other studies have revealed that after oral administration of crocetin (100 mg/kg) for 90 min, the brain concentration was approximately 2.43 nmol/g (Wong et al., 2020). Therefore, future investigations need to examine whether crocetin can cross the BBB and determine the effect of the crocetin configuration on BBB permeation.

It is well-established that crocetin can pass through the intestinal barrier. Several studies have shown that crocetin can be rapidly absorbed into the blood *via* the gastrointestinal tract, reaching peak plasma concentration for a short period (Colapietro et al., 2019). In a study by Christodoulou et al., following oral and intravenous administration of an aqueous saffron extract (60 mg/kg) to C57/Bl6J mice, crocetin (derived from *in vivo* crocin hydrolysis) tissue levels were measured using the HPLC-PDA method, and non-compartmental pharmacokinetic analysis was performed. Crocetin was extensively distributed in the liver and kidney, the main organs for crocetin biotransformation and elimination. The levels of crocetin could not be detected in the lungs and heart, possibly because of rapid glucuronidation to form its conjugated forms (Christodoulou et al., 2019). However, Du et al. showed that orally administered crocetin is widely distributed in the body. The crocetin concentration in tissues, ranging from high to low, was found to occur in the following order: liver, lung, ovary, kidney, fat, and testis (Du et al., 2004). Additional research is needed to determine the distribution of crocetin in the body.

Crocetin-γ-cyclodextrin was administered to SD rats *via* intravenous and intraperitoneal injections. Crocetin-γ-cyclodextrin can enter the brain by crossing the BBB and is distributed in the stomach, large intestine, small intestine, liver, spleen, kidney, lung, and heart. The bioavailability of intravenous injection was greater than that of intraperitoneal injection, and the drug concentration in each tissue reached a maximum after 5 min of intravenous injection. After 4 h, nearly no residual drug could be detected, and the metabolic rate was relatively elevated (Wong et al., 2020). In a study examining the inhibitory effect of crocetin on proliferative vitreoretinopathy in rabbits, 0.4 μmol crocetin was administered as an intravitreal injected into PVR rabbit eyes. The half-life of crocetin was determined as 4.231 h by HPLC. One hour after intravitreal injection, the C_max_ of crocetin was 36.77 ± 3.39 μg/ml (Wang S. L. et al., 2018).

### 6.3 Metabolism

The whole *in vitro* digestion process (salivary, stomach, and duodenal steps) designed by Almodóvar et al. showed no increase in the crocetin concentration when compared with the original composition of saffron (Almodóvar et al., 2020). The results indicated that saffron extract was not metabolized into crocetin in these parts of the body.

Almodóvar et al. speculated that crocetin formation in the blood could be attributed to the presence of enzymes in the epithelial cells of the gastrointestinal tract, which can hydrolyze the crocin isomers (Almodóvar et al., 2020). Enzymes of gastrointestinal tract epithelial cells include esterase or β-glycosidase (Németh et al., 2003).

Lautenschläger et al. also believed that the intestinal deglycosylation of different types of crocin was primarily attributed to enzymatic processes in the epithelial cells and only to a very small extent to deglycosylation by the fecal microbiome (Lautenschläger et al., 2015). *In vivo* and *in vitro* experiments by Zhang et al. have shown that crocin can be deglycosylated to crocetin in the intestinal content of normal rats; however, this transformation did not occur in pseudo-germ-free (pGF) rats, suggesting that the intestinal microbiota plays a key role transforming crocin into crocetin. A possible explanation might be that crocin was not absorbed by enterocytes following oral administration, and a considerable portion of crocin was retained in the intestinal tract, where it could be directly metabolized into crocetin by intestinal flora (Zhang Y. et al., 2019). Further studies are needed to confirm the precise intestinal section involved. Moreover, crocetin is metabolized to ester-type glucuronides in the liver or gut mucosa after oral administration. This form of crocetin showed stronger stability in plasma and could be considered a bioactive molecule or a carrier to deliver crocetin to target tissues (Mh and Hhb 2019).

Lautenschläger et al. used mouse intestinal tissue and fecal homogenate for metabolism experiments. Fecal bacteria degraded the apocarotenoid backbone into smaller alkyl units, which did not demonstrate any typical ultraviolet (UV) absorption peaks of crocetin. Additional liquid chromatography (LC)-MS studies indicated the absence of specific degradation products (data not shown) (Lautenschläger et al., 2015).

### 6.4 Excretion

Crocetin was not excreted in the urine or feces after intravenous administration. Orally administered crocetin is mainly excreted in feces (Zhang X. et al., 2019). Umigai et al. showed that crocetin was eliminated from human plasma with a half-life (T_1/2_) ranging between 6.1 and 7.5 h (7.5, 15, and 22.5 mg in one-weekly interval) (Umigai et al., 2011). Zhang et al. reported that the plasma T_1/2_ was calculated from 1.640 to 1.671 h, as detected by HPLC after oral administration of crocetin (25 and 100 mg/kg) in rats. After intravenous administration, the T_1/2_ of crocetin (5 mg/kg) was 1.914 h (Zhang 2017).

Few studies have examined the pharmacokinetics of crocetin. Additional data are crucial to better understand the effectiveness and dose-response relationship of crocetin, and it is important to determine the doses, directions for use, and further development of crocetin.

## 7 Drug Safety

Given the extensive range of pharmacological activities, the safety of crocetin has been widely examined.

### 7.1 Clinical Research

In a clinical trial assessing the effect of crocetin on sleep quality, subjects were given crocetin capsules (7.5 mg) once daily for 14 days. No crocetin-induced adverse reactions were observed (Kuratsune et al., 2010).

In a study by Mori et al., the effect of crocetin was assessed in children with myopia. The subjects were given soft capsules containing 7.5 mg of crocetin, taken orally once a day for 24 weeks. No adverse reactions associated with crocetin were reported during the clinical study. All adverse events reported were unrelated to crocetin administration (Mori et al., 2019). In a clinical study evaluating the pharmacokinetics of crocetin, 10 healthy volunteers had no significant adverse events after oral administration of 22.5 mg crocetin (Umigai et al., 2011). In another clinical study evaluating crocetin for physical fatigue relief, healthy volunteers were orally administered crocetin (15 mg) for 8 days (Wang S. L. et al., 2018). Similar to previous studies, no significant discomfort was observed in healthy volunteers at the end of the study period. A phase II clinical trial assessing intravenous crocetin (0.25 mg/kg) for acute stroke was approved by the Food and Drug Administration (Abedimanesh et al., 2019).

### 7.2 Animal/Cell Research

Related animal experiments showed that the LD_50_ of crocetin was 20.7 g/kg body weight following oral administration (Abdullaev 2002).

Crocetin exhibited selective toxicity against cancer cells and may be effective in cancer prevention. However, the cytotoxicity of crocetin on normal cells is negligible, and oral administration is non-toxic (Milajerdi et al., 2016). Jagadeeswaran et al. demonstrated that crocetin (5–20 μg/ml) had selective cytotoxic effects against human rhabdomyosarcoma cells, with poor cytotoxic effects on normal cells when compared with cisplatin-mediated cytotoxicity (Jagadeeswaran et al., 2000). Some studies have shown that 50–100 mg/kg crocetin could protect gastric cancer tissues in rats and had no cytotoxic effect in normal rats (Bathaie et al., 2013a). In addition, studies have shown that the percentage of cytotoxic effect of crocetin beta-D-glucosyl ester (31.25–1,000 mg/ml) ranged between 18.5 and 61.57% in MCF-7 cells when examined at different concentrations (Mam et al., 2020). Previous studies have shown that continuous doses exceeding 10 g can be toxic and cause uterine stimulation and miscarriage during pregnancy (Wüthrich et al., 2010). Martin et al. examined the teratogenic effect of crocetin in frog embryos, and the results showed that a high concentration of crocetin (200 μmol) exerted a teratogenic effect; however, the teratogenic effect was considerably less than that of all-trans-methyl acid (Martin et al., 2002).

## 8 Drug Formulation and Preparation Research

Low water solubility, poor oral absorption, and low bioavailability are key characteristics of crocetin. Therefore, the drug dosage form can be modified to improve these unfavorable features (Carmelo et al., 2018).

### 8.1 Crocetin injection

Zhang et al. patented the production of crocetin injections. The raw material crocetin and auxiliary materials such as propylene glycol were mixed to dissolve the raw crocetin material. Subsequently, injection water and activated carbon were added. A qualified crocetin injection was then prepared. After filtration, filling, lamp inspection, and packaging, a qualified crocetin injection was prepared. The crocetin injection had good stability, simple preparation, and low cost. The bioavailability of crocetin can be improved by formulating a crocetin injection (Zhang et al., 2011). Another modification involves the preparation of crocetin salt injections. The injection was obtained by dissolving crocetin salt and sodium chloride in water, which can improve the bioavailability and treatment effects of crocetin (Wang and Li 2015).

### 8.2 Nanoparticle Drug

NP-based drug delivery systems are promising new drug delivery systems that can improve drug delivery efficiency by improving the pharmacokinetics and overcoming the shortcomings of native drugs (Feng 2006).

Neyshaburinezhad et al. encapsulated crocetin into poly (lactic-co-glycolic acid) nanoparticles using a single emulsion-solvent evaporation method. The particle size was determined as 239.8 ± 9 nm. The entrapment efficiency and loading capacity of crocetin NPs were approximately 79 ± 3% and 4.9 ± 0.2%, respectively (Neyshaburinezhad et al., 2019). Yang et al. prepared a nano-formulation of crocetin (CT-PLGA-NPs) using a double emulsion evaporation technique, which employed Span 60 and Tween 80 as the internal and external aqueous phases, respectively, and polyvinyl alcohol (1%) was used to stabilize the external aqueous phase (Yang 2019). In addition, Langroodi et al. used the solvent evaporation/double emulsion method to load crocetin and doxorubicin into PLGA NPs. The prepared NPs exhibited a particle size of 200–300 nm, and the drug loading efficiencies of crocetin and doxorubicin were 65 and 54%, respectively. In addition, the prepared NPs inhibited the growth of MCF-7 tumor cells more effectively (Langroodi et al., 2017).

Pradhan et al. prepared crocetin-loaded lipid NPs using glycerol monooleate (GMO), a synthetic, non-toxic, biocompatible, biodegradable material as the carrier material. The physical characteristics of crocetin-loaded NPs were obtained on measurement: particle size, 119 ± 4 nm; zeta potential, 18.3 ± 4.21 mV; polydispersity index, 0.426. The crocetin-loaded NPs exhibited an encapsulation efficiency of 80%. The low PDI indicated that the particles were uniformly distributed, and the negative zeta potential was conducive to the mutual exclusion of the formulations, which ensured particle stability and prevented particle aggregation (Jyotsnarani et al., 2018). Photodynamic therapy (PDT) is a new method for treating tumor diseases using a photosensitizer, commonly known as indocyanine green (ICG). NPs were used with ICG to overcome the high cytotoxicity at higher concentrations and instability in aqueous media. Sazgarnia et al. loaded crocetin into PLGA NPs to improve the efficacy of PDT with ICG against MCF-7 cells. Accordingly, PLGA-CRT NPs combined with ICG could improve PDT results more effectively. This method afforded low cytotoxicity for treating breast cancer (Sazgarnia et al., 2021).

Wong et al. encapsulated crocetin into γ-cyclodextrin using an ultrasonic method. Crocetin-γ-cyclodextrin inclusion complexes demonstrated good water solubility and were found to be suitable for intravenous injection. Based on the pharmacokinetics and biodistribution, the crocetin-γ-cyclodextrin inclusion complex can improve the crocetin bioavailability and promote BBB crossing, which is beneficial for treating neurosystemic diseases, such as AD (Wong et al., 2020).

Puglia et al. used softisan 100 (hydrogenated coco-glycerides) as solid lipid matrixes and solvent diffusion technology to prepare crocetin solid lipid NPs, exhibiting an average diameter of 280 nm and zeta potential value of −17.8 mV, implying that the NPs have good long-term stability. The nanodispersion displayed good stability, with an encapsulation efficiency of 80% (Carmelo et al., 2018). Current treatment methods have improved the impaired oxygen transportation in acute respiratory distress syndrome, which is beneficial for treating patients with severe respiratory complications of coronavirus disease (COVID-19). A phase I/II clinical trial of the liposomal nanocarrier encapsulating *trans*-crocetin enhanced the oxygenation of vascular tissue, indicating the potential to treat respiratory distress syndrome due to COVID-19. In addition, the liposomal formulations could increase the reoxygenation properties of free *trans*-crocetin in endothelial cells from 30 min to 48 h. The clinical experimental results showed that the proportion of partial arterial pressure of oxygen (O_2_) to the inspired fraction of O_2_ (PaO_2_/FiO_2_ proportion) improved by ≥25% in patients with acute respiratory distress syndrome under artificial respiratory support. Accordingly, liposomal encapsulation of *trans*-crocetin enhanced oxygenation in patients with COVID-19-related acute respiratory distress syndrome on mechanical ventilation (Mertes et al., 2021).

### 8.3 Microencapsulation

To increase the stability of crocetin, Zhou et al. used gum acacia as wall material and spray-drying technology to microencapsulate crocetin; the microencapsulation rate reached 85.03%. The deterioration rate was consistent with the first kinetic model (Hui et al., 2013).

### 8.4 Solid Dispersion Sustained Release Tablets

Song et al. prepared crocetin solid dispersion using the solvent method and PVPK30 as the carrier material; the optimal dosage ratio of crocetin and PVPK30 was 1:4. Drug release was significantly higher from the prepared solid dispersion than from the bulk drug. Based on previous experiments, HPMC-K4M and HPMC-K15M (6:4) were used as sustained-release skeleton materials, and MCC was used as the filler to prepare crocetin solid dispersion sustained-release tablets. The drug content of the prepared sustained-release tablets was 98.02%. The release rates at 2, 6, 12, and 24 h were 8, 22, 27, and 34%, respectively, indicating good sustained-release effects *in vitro*. Finally, the pharmacokinetics of crocetin solid dispersion sustained-release tablets were assessed in beagle dogs. For the bulk drug, solid dispersion tablet, and sustained-release tablets, the AUC_0-24_ values were 33.74, 39.64, and 86.06 μg·h/ml, the C_max_ values were 3.79, 10.95, and 11.05 μg·ml^−1^, T_max_ values were 2, 0.5, and 4 h, respectively. The average bioavailability of sustained-release tablets was 255.07 and 217.10% when compared with bulk drugs and solid dispersions, respectively (Zhong 2014).

## 9 Conclusion

Saffron, a traditional medicinal agent with multiple functions, has been widely assessed in research and development. The primary active ingredients of saffron mediating pharmacological effects are crocin and crocetin. Crocetin is an aglycone of crocin naturally occurring in saffron and produced in biological systems by hydrolysis of crocin as a bioactive metabolite. Therefore, the application of crocetin is worthy of attention.

1) The scarcity and expense of saffron will greatly limit the application of crocetin. Extracting and producing crocetin from other natural sources is an important way to overcome this shortcoming. In addition to saffron, *G. jasminoides* also contains crocin and crocetin, and may be an economical alternative to saffron. In the following research, we are committed to developing eco-friendly, cost-effective, convenient, and efficient methods for extracting crocetin.
2) The structure of crocetin contains two carboxyl groups. Therefore, the oxidation and esterification characteristics of carboxyl groups can be used to modify the crocetin structure, thus synthesizing different crocetin derivatives. This is expected to improve pharmacological activity and expand the scope of action of crocetin. However, crocetin dissolution in most organic solutions is poor, which increases the difficulty of chemical synthesis reactions. In subsequent research, we need to focus on this point.
3) Based on the structure of polyunsaturated conjugated acids, the excellent antioxidant activity can explain the diverse pharmacological properties of crocetin. Crocetin researches focus on the evaluation of pharmacological properties, and the studies on the mechanisms of action should be strengthened.
4) Although crocetin showed therapeutic effects against cardiovascular diseases, cancer, and nervous system diseases *in vivo* and *in vitro,* evidence from clinical trials remains insufficient. To further clarify its pharmacological effects, clinical research should be undertaken in subsequent investigations.
5) Research on new crocetin formulations has demonstrated considerable benefits. Systematic drug delivery, such as solid lipid NPs, microencapsulation, and solid dispersion sustained-release tablets improved drug solubility, absorption, and bioavailability. In subsequent research on crocetin, the development of new dosage forms and preparations has broad research implications.
6) So that crocetin can be used clinically in the future, further drug safety research is warranted, particularly to examine the possible toxic effects of crocetin during long-term administration.

## References

[B1] Abdullaev F. I. (2002). Cancer Chemopreventive and Tumoricidal Properties of Saffron (Crocus Sativus L.). Exp. Biol. Med. (Maywood) 227 (1), 20–25. 10.1177/153537020222700104 11788779

[B2] Abedimanesh S. Bathaie S. Z. Ostadrahimi A. Asghari Jafarabadi M. Taban Sadeghi M. (2019). The Effect of Crocetin Supplementation on Markers of Atherogenic Risk in Patients with Coronary Artery Disease: a Pilot, Randomized, Double-Blind, Placebo-Controlled Clinical Trial. Food Funct. 10 (4), 7461–7475. 10.1039/c9fo01166h 31667483

[B3] Ahmad A. S. Ansari M. A. Ahmad M. Saleem S. Yousuf S. Hoda M. N. (2005). Neuroprotection by Crocetin in a Hemi-Parkinsonian Rat Model. Pharmacol. Biochem. Behav. 81 (4), 805–813. 10.1016/j.pbb.2005.06.007 16005057

[B4] Ahn J. H. Hu Y. Hernandez M. Kim J. R. (2011). Crocetin Inhibits Beta-Amyloid Fibrillization and Stabilizes Beta-Amyloid Oligomers. Biochem. Biophys. Res. Commun. 414 (1), 79–83. 10.1016/j.bbrc.2011.09.025 21945434

[B5] Almodóvar P. Briskey D. Rao A. Prodanov M. Inarejos-García A. M. (2020). Bioaccessibility and Pharmacokinetics of a Commercial Saffron (Crocus Sativus L.) Extract( Crocus Sativus L.) Extract. Evid. Based Complement. Alternat Med. 2020 (11), 1575730–1575738. 10.1155/2020/1575730 32089715 PMC7013346

[B6] Ando H. Kondoh H. Ichihashi M. Hearing V. J. (2007). Approaches to Identify Inhibitors of Melanin Biosynthesis via the Quality Control of Tyrosinase. J. Invest. Dermatol. 127 (4), 751–761. 10.1038/sj.jid.5700683 17218941

[B7] Azarhazin E. Izadyar M. Housaindokht M. R. (2017). Molecular Dynamic Simulation and DFT Study on the Drug-DNA Interaction; Crocetin as an Anti-cancer and DNA Nanostructure Model. J. Biomol. Struct. Dyn. 36 (4), 1063–1074. 10.1080/07391102.2017.1310060 28330413

[B8] Bathaie S. Z. Hoshyar R. Miri H. Sadeghizadeh M. (2013a). Anticancer Effects of Crocetin in Both Human Adenocarcinoma Gastric Cancer Cells and Rat Model of Gastric Cancer. Biochem. Cel Biol 91 (6), 397–403. 10.1139/bcb-2013-0014 24219281

[B9] Bathaie S. Z. Miri H. Mohagheghi M. A. Mokhtari-Dizaji M. Shahbazfar A. A. Hasanzadeh H. (2013b). Saffron Aqueous Extract Inhibits the Chemically-Induced Gastric Cancer Progression in the Wistar Albino Rat. Iran J. Basic Med. Sci. 16 (1), 27–38. 10.1016/j.drudis.2012.08.007 23638290 PMC3637902

[B10] Bie X. Chen Y. Zheng X. Dai H. (2011). The Role of Crocetin in protection Following Cerebral Contusion and in the Enhancement of Angiogenesis in Rats. Fitoterapia 82 (7), 997–1002. 10.1016/j.fitote.2011.06.001 21741458

[B11] Boyd-Kimball D. Sultana R. Mohmmad-Abdul H. Butterfield D. A. (2005). Neurotoxicity and Oxidative Stress in D1M-Substituted Alzheimer's A Beta(1-42): Relevance to N-Terminal Methionine Chemistry in Small Model Peptides. Peptides 26 (4), 665–673. 10.1016/j.peptides.2004.11.001 15752582

[B12] Cai H. L. Tang S. Y. Zhou J. Liu Y. H. (2012). Effect of Down-Regulation of Crocetin on Expression of LOX-1 in Atherosclerosis Rats. China Trop. Med. (01), 12–14. 10.13604/j.cnki.46-1064/r.2012.01.015

[B13] Cai J. Yi F. F. Bian Z. Y. Shen D. F. Yang L. Yan L. (2009). Crocetin Protects against Cardiac Hypertrophy by Blocking MEK-Erk1/2 Signalling Pathway. J. Cel Mol Med 13 (8B), 909–925. 10.1111/j.1582-4934.2008.00620.x PMC3823407 19413885

[B14] Cao W. Cui J. Li S. Zhang D. Guo Y. Li Q. (2017). Crocetin Restores Diabetic Endothelial Progenitor Cell Dysfunction by Enhancing NO Bioavailability via Regulation of PI3K/AKT-eNOS and ROS Pathways. Life Sci. 181, 9–16. 10.1016/j.lfs.2017.05.021 28528862

[B15] Cardone L. Castronuovo D. Perniola M. Cicco N. Candido V. (2020). Saffron (Crocus Sativus L.), the king of Spices: An Overview. Scientia Horticulturae 272, 109560. 10.1016/j.scienta.2020.109560

[B16] Carmona M. Zalacain A. Sánchez A. M. Novella J. L. Alonso G. L. (2006). Crocetin Esters, Picrocrocin and its Related Compounds Present in Crocus Sativus Stigmas and Gardenia Jasminoides Fruits. Tentative Identification of Seven New Compounds by LC-ESI-MS. J. Agric. Food Chem. 54 (3), 973–979. 10.1021/JF052297W 16448211

[B17] Chalatsa I. Arvanitis D. A. Koulakiotis N. S. Giagini A. Skaltsounis A. L. Papadopoulou-Daifoti Z. (2019). The Crocus Sativus Compounds Trans-crocin 4 and Trans-crocetin Modulate the Amyloidogenic Pathway and Tau Misprocessing in Alzheimer Disease Neuronal Cell Culture Models. Front. Neurosci. 13, 249. 10.3389/fnins.2019.00249 30971876 PMC6443833

[B18] Chen B. Hou Z.-H. Dong Z. Li C.-D. (2015). Crocetin Downregulates the Proinflammatory Cytokines in Methylcholanthrene-Induced Rodent Tumor Model and Inhibits COX-2 Expression in Cervical Cancer Cells. Biomed. Res. Int. 2015, 1–5. 10.1155/2015/829513 PMC4385625 25874230

[B19] Cheng Q. L. Li H. L. Zhang D. Y. (2010). Study on Anti-arrhythmia Effect of Crocetin. Med. J. Chin. People's Liberation Army 35 (04), 395–397. 10.4268/cjcmm20100311

[B20] Christodoulou E. GrafakouMaria-Eleni M. E. Skaltsa E. Kadoglou N. Kostomitsopoulos N. Valsami G. (2019). Preparation, Chemical Characterization and Determination of Crocetin's Pharmacokinetics after Oral and Intravenous Administration of Saffron (Crocus Sativus L.) Aqueous Extract to C57/BL6J Mice. J. Pharm. Pharmacol. 71 (5), 753–764. 10.1111/jphp.13055 30575029

[B21] Chryssanthi D. G. Karamanos N. K. Cordopatis P. Lamari F. N. Lamari F. (2010). Crocetin Inhibits Invasiveness of MDA-MB-231 Breast Cancer Cells via Downregulation of Matrix Metalloproteinases. Planta Med. 77, 146–151. 10.1055/s-0030-1250178 20803418

[B22] Chu Y. Gao J. Niu J. Huang Y. F. Chen M. Wang M. Z. (2018). Synthesis, Characterization and Inhibitory Effects of Crocetin Derivative Compounds in Cancer and Inflammation. Biomed. Pharmacother. 98, 157–164. 10.1016/j.biopha.2017.12.018 29253763

[B23] Colapietro A. Mancini A. D'Alessandro A. M. Festuccia C. (2019). Crocetin and Crocin from Saffron in Cancer Chemotherapy and Chemoprevention. Acamc 19, 38–47. 10.2174/1871520619666181231112453 30599111

[B24] D'Alessandro A. M. Mancini A. Lizzi A. R. De Simone A. Marroccella C. E. Gravina G. L. (2013). Crocus Sativus Stigma Extract and its Major Constituent Crocin Possess Significant Antiproliferative Properties against Human Prostate Cancer. Nutr. Cancer 65, 930–942. 10.1080/01635581.2013.767368 23909737

[B25] Dhar A. Mehta S. Dhar G. Dhar K. Banerjee S. Van Veldhuizen P. (2009). Crocetin Inhibits Pancreatic Cancer Cell Proliferation and Tumor Progression in a Xenograft Mouse Model. Mol. Cancer Ther. 8 (2), 315–323. 10.1158/1535-7163.MCT-08-0762 19208826

[B26] Diao S. L. Sun J. W. Ma B. X. Li X. M. Wang D. (2018). Influence of Crocetin on High-Cholesterol Diet Induced Atherosclerosis in Rats via Anti-oxidant Activity Together with Inhibition of Inflammatory Response and P38 MAPK Signaling Pathway. Saudi J. Biol. Sci. 25, 493–499. 10.1016/j.sjbs.2016.11.005 29692651 PMC5911641

[B27] Ding F. Liu F. Shao W. Chu J. Wu B. He B. (2018). Efficient Synthesis of Crocins from Crocetin by a Microbial Glycosyltransferase from Bacillus Subtilis 168. J. Agric. Food Chem. 66 (44), 11701–11708. 10.1021/acs.jafc.8b04274 30350978

[B28] Ding J. Su J. Zhang L. Ma J. (2015). Crocetin Activates Foxp3 through TIPE2 in Asthma-Associated Treg Cells. Cell Physiol Biochem 37 (6), 2425–2433. 10.1159/000438595 26646898

[B29] Dong N. Dong Z. Chen Y. Gu X. (2020). Crocetin Alleviates Inflammation in MPTP-Induced Parkinson's Disease Models through Improving Mitochondrial Functions. Parkinson's Dis. 2020, 1–9. 10.1155/2020/9864370 PMC7569465 33101635

[B30] D.Pawar N. S.Panchal S. J. K. Shahmalak T. Gadgoli C. Gadgoli C. (2015). Crocin Rich Extract of Nyctanthes Arbor-Tristis Flower Calyx Induces Anti-angiogenic Activity. Npj 06, 1. 10.2174/2210315506666151208212552

[B31] Drechsler M. Megens R. T. van Zandvoort M. Weber C. Soehnlein O. (2010). Hyperlipidemia-triggered Neutrophilia Promotes Early Atherosclerosis. Circulation 122 (18), 1837–1845. 10.1161/CIRCULATIONAHA.110.961714 20956207

[B32] Du P. Qian Z. Y. Yu W. P. Xing Y. (2004). Study on Pharmacokinetics and Tissue Distribution of Crocetin in Rats by RP-HPLC. Chin. J. Pharm. Anal. 24 (02), 149–153.

[B33] Eidenberger T. (2010). Hydrolysate of Crocin.

[B34] Ekremoglu O. Koc A. (2021). The Role of SIRT5 and p53 Proteins in the Sensitivity of Colon Cancer Cells to Chemotherapeutic Agent 5-Fluorouracil . Res. Square. 10.21203/rs.3.rs-222345/v1 34279763

[B35] Elgazar A. F. Rezq A. A. Bukhari H. (2013). Anti-Hyperglycemic Effect of Saffron Extract in Alloxan-Induced Diabetic Rats. Eur. J. Biol. Sci. 5 (1), 14–22. 10.5829/idosi.ejbs.2013.5.1.7224

[B36] Erfanparast A. Tamaddonfard E. Henareh-Chareh F. (2020). Central H2 Histaminergic and Alpha-2 Adrenergic Receptors Involvement in Crocetin-Induced Antinociception in Orofacial Formalin Pain in Rats. Vet. Res. Forum 11, 229–234. 10.30466/vrf.2018.83779.2101 33133459 PMC7597797

[B37] Fang Z. K. Wang J. X. (2007). Preparation of Crocetin and Crocetin Dimethyl Ester and Research of Their Antioxidative Activities. Chengdu, China: Natural Product Research and Development, 280–282. 10.16333/j.1001-6880.2007.02.026

[B38] Farkhondeh T. T. Samarghandian S. Samini F. Sanati A. R. (2018). Protective Effects of Crocetin on Depression-like Behavior Induced by Immobilization in Rat. CNS Neurol. Disord. Drug Targets 17 (5), 361–369. 10.2174/1871527317666180515120212 29766828

[B39] Feng S. S. (2006). Nanoparticle-based Drug Delivery System.

[B40] Fernández-Albarral J. A. de Hoz R. Ramírez A. I. López-Cuenca I. Salobrar-García E. Pinazo-Durán M. D. (2020). Beneficial Effects of Saffron (Crocus Sativus L.) in Ocular Pathologies, Particularly Neurodegenerative Retinal Diseases. Neural Regen. Res. 15 (8), 1408–1416. 10.4103/1673-5374.274325 31997799 PMC7059587

[B41] Fernández-Albarral J. A. Ramírez A. I. de Hoz R. López-Villarín N. Salobrar-García E. López-Cuenca I. (2019). Neuroprotective and Anti-inflammatory Effects of a Hydrophilic Saffron Extract in a Model of Glaucoma. Int. J. Mol. Sci. 20, 1–22. 10.3390/ijms20174110 PMC6747458 31443568

[B42] Festuccia C. Mancini A. Gravina G. L. Scarsella L. Llorens S. Alonso G. L. (2014). Antitumor Effects of Saffron-Derived Carotenoids in Prostate Cancer Cell Models. Biomed. Res. Int. 2014 (5), 1–12. 10.1155/2014/135048 PMC4037572 24900952

[B43] Gao K. Guo H. X. Liu L. M. Ding Y. Q. (2016). Hepatoprotective Effect of Crocetin on Paraquat Poisoned Rats. Chin. Crit. Care Med. 28 (10), 876–880. 10.3760/cma.j.issn.2095-4352.2016.10.003

[B44] Gao K. Liu F. Chen X. Chen M. Deng Q. Zou X. (2019). Crocetin Protects against Fulminant Hepatic Failure Induced by lipopolysaccharide/D-Galactosamine by Decreasing Apoptosis, Inflammation and Oxidative Stress in a Rat Model. Exp. Ther. Med. 18, 3775–3782. 10.3892/etm.2019.8030 31616509 PMC6781807

[B45] Granchi C. Fortunato S. Meini S. Rizzolio F. Caligiuri I. Tuccinardi T. (2017). Characterization of the Saffron Derivative Crocetin as an Inhibitor of Human Lactate Dehydrogenase 5 in the Antiglycolytic Approach against Cancer. J. Agric. Food Chem. 65 (28), 5639–5649. 10.1021/acs.jafc.7b01668 28643510

[B46] Guan X. Shao P. Li X. (2021). Chemoprotective Effect of Crocetin against 1,2 Dimethyl Hydrazine Induced Colorectal Cancer in Albino Wistar Rats through Antioxidant Pathway. Phcog Mag. 17, 360–366. 10.4103/pm.pm_311_20

[B47] Guo H. Gao K. Zou X. Deng Q. Chen M. Liu F. (2018). Crocetin Promotes Autophagy in Injured Rat Hepatocytes Induced by Lipopolysaccharide and D-Galactosamine *In Vitro* . Nan Fang Yi Ke Da Xue Xue Bao 38 (09), 1121–1125. 10.12122/j.issn.1673-4254.2018.09.16 30377103 PMC6744181

[B48] Gutheil W. G. Reed G. Ray A. Anant S. Dhar A. (2012). Crocetin: an Agent Derived from Saffron for Prevention and Therapy for Cancer. Curr. Pharm. Biotechnol. 13 (1), 173–179. 10.2174/138920112798868566 21466430 PMC4461363

[B49] Hashemi M. Hosseinzadeh H. (2019). A Comprehensive Review on Biological Activities and Toxicology of Crocetin. Food Chem. Toxicol. 130, 44–60. 10.1016/j.fct.2019.05.017 31100302

[B50] Hashemi S. A. Bathaie S. Z. Mohagheghi M. A. (2020). Crocetin and Crocin Decreased Cholesterol and Triglyceride Content of Both Breast Cancer Tumors and Cell Lines. Avicenna J. Phytomed 10, 384–397. 10.22038/AJP.2019.14372 32850295 PMC7430959

[B51] Hashemi S. A. Karami M. Bathaie S. Z. (2020). Saffron Carotenoids Change the Superoxide Dismutase Activity in Breast Cancer: *In Vitro*, *In Vivo* and In Silico Studies. Int. J. Biol. Macromolecules 158, 845–853. 10.1016/j.ijbiomac.2020.04.063 32360463

[B52] Hashemi-Shahri S. H. Golshan A. Mohajeri S. A. Baharara J. Amini E. Salek F. (2018). ROS-scavenging and Anti-tyrosinase Properties of Crocetin on B16F10 Murine Melanoma Cells. Anticancer Agents Med. Chem. 18 (7), 1064–1069. 10.2174/1871520618666171213143455 29237384

[B53] Hashemi-Shahri S. H. Golshan A. MohajeriBaharara S. A. Baharara J. Amini E. Salek F. (2018). ROS-scavenging and Anti-tyrosinase Properties of Crocetin on B16F10 Murine Melanoma Cells. Anticancer Agents Med. Chem. 18 (7), 1064–1069. 10.2174/1871520618666171213143455 29237384

[B54] He B. F. Ding F. Y. Liu F. Shao W. M. Wang G. J. (2017). Glucosyltransferase and its Application in Synthesizing Crocetin Glucose Ester.

[B55] He K. Si P. Wang H. Tahir U. Chen K. Xiao J. (2014). Crocetin Induces Apoptosis of BGC-823 Human Gastric Cancer Cells. Mol. Med. Rep. 9 (2), 521–526. 10.3892/mmr.2013.1851 24337515

[B56] Herman A. (2019). Probiotics Supplementation in Prophylaxis and Treatment of Depressive and Anxiety Disorders - a Review of Current Research. Psychiatr. Pol. 53 (02), 459–473. 10.12740/PP/92392 31317970

[B57] Higashino S. Sasaki Y. Giddings J. C. Hyodo K. Sakata S. F. Matsuda K. Horikawa Y. Yamamoto J. (2014). Crocetin, a Carotenoid from Gardenia Jasminoides Ellis, Protects against Hypertension and Cerebral Thrombogenesis in Stroke-Prone Spontaneously Hypertensive Rats Prone Spontaneously Hypertensive Rats. Phytother Res. 28, 1315–1319. 10.1002/ptr.5130 24550159

[B58] Himori N. Inoue Yanagimachi M. Omodaka K. Shiga Y. Tsuda S. Kunikata H. (2021). The Effect of Dietary Antioxidant Supplementation in Patients with Glaucoma. Opth 15, 2293–2300. 10.2147/OPTH.S314288 PMC8183457 34113073

[B59] Inoue E. Shimizu Y. Masui R. Hayakawa T. Tsubonoya T. Hori S. (2018). Effects of Saffron and its Constituents, crocin-1, Crocin-2, and Crocetin on α-synuclein Fibrils. J. Nat. Med. 72, 274–279. 10.1007/s11418-017-1150-1 29147836

[B60] Ishizuka F. Shimazawa M. Umigai N. Ogishima H. Nakamura S. Tsuruma K. (2013). Crocetin, a Carotenoid Derivative, Inhibits Retinal Ischemic Damage in Mice. Eur. J. Pharmacol. 703 (1-3), 1–10. 10.1016/j.ejphar.2013.02.007 23428630

[B61] Jagadeeswaran R. Thirunavukkarasu C. Gunasekaran P. Ramamurty N. Sakthisekaran D. (2000). *In Vitro* studies on the Selective Cytotoxic Effect of Crocetin and Quercetin. Fitoterapia 71 (4), 395–399. 10.1016/S0367-326X(00)00138-6 10925010

[B62] Kalalinia F. Ghasim H. Amel Farzad S. Pishavar E. Ramezani M. Hashemi M. (2018). Comparison of the Effect of Crocin and Crocetin, Two Major Compounds Extracted from Saffron, on Osteogenic Differentiation of Mesenchymal Stem Cells. Life Sci. 208, 262–267. 10.1016/j.lfs.2018.07.043 30048694

[B63] Kanakis C. D. Tarantilis P. A. Tajmir-Riahi H. A. Polissiou M. G. (2007). Crocetin, Dimethylcrocetin, and Safranal Bind Human Serum Albumin: Stability and Antioxidative Properties. J. Agric. Food Chem. 55 (3), 970–977. 10.1021/jf062638l 17263501

[B64] Karimi P. Gheisari A. Gasparini S. J. Baharvand H. Shekari F. Satarian L. (2020). Crocetin Prevents RPE Cells from Oxidative Stress through Protection of Cellular Metabolic Function and Activation of ERK1/2. Int. J. Mol. Sci. 21, 2949–2973. 10.3390/ijms21082949 PMC7215651 32331354

[B65] Kazi H. A. Qian Z. (2009). Crocetin Reduces TNBS-Induced Experimental Colitis in Mice by Downregulation of NFkB. Saudi J. Gastroenterol. 15 (3), 181–187. 10.4103/1319-3767.54750 19636180 PMC2841418

[B66] Khorasany A. R. Hosseinzadeh H. (2016). Therapeutic Effects of Saffron (Crocus Sativus L.) in Digestive Disorders: a Review. Iran J. Basic Med. Sci. 19 (5), 455–469. 27403251 PMC4923465

[B67] Kim S. H. Lee J. M. Kim S. C. Park C. B. Lee P. C. (2014). Proposed Cytotoxic Mechanisms of the Saffron Carotenoids Crocin and Crocetin on Cancer Cell Lines. Biochem. Cel Biol 92 (2), 105–111. 10.1139/bcb-2013-0091 24697694

[B68] Kong Y. Kong L. P. Luo T. Li G. W. Jiang W. Li S. (2014). The Protective Effects of Crocetin on Aβ₁₋₄₂-Induced Toxicity in Ht22 Cells. CNS Neurol. Disord. Drug Targets 13, 1627–1632. 10.2174/1871527313666140806125410 25106634

[B69] Kumar S. Gupta S. K. (2019). A Review on Therapeutic Potentials of Crocetin-A Carotenoid Derived from Saffron.

[B70] Kuratsune H. Umigai N. Takeno R. Kajimoto Y. Nakano T. (2010). Effect of Crocetin from Gardenia Jasminoides Ellis on Sleep: A Pilot Study. Phytomedicine 17 (11), 840–843. 10.1016/j.phymed.2010.03.025 20537515

[B71] Lam T. T. Abler A. S. Kwong J. M. Tso M. O. (1999). N-methyl-D-aspartate (NMDA)--induced Apoptosis in Rat Retina. Invest. Ophthalmol. Vis. Sci. 40 (10), 2391–2397. 10476807

[B72] Langroodi F. A. Hafezi Ghahestani Z. Alibolandi M. Ebrahimian M. Hashemi M. (2017). Improvement of Antitumor Activity of Doxorubicin by Co-formulation of Crocetin and Doxorubicin in PLGA Nanoparticles. J. Cell Immunother. 3 (1), 8. 10.1016/j.jocit.2017.04.010

[B73] Lautenschläger M. Lechtenberg M. Sendker J. Hensel A. (2014). Effective Isolation Protocol for Secondary Metabolites from Saffron: Semi-preparative Scale Preparation of Crocin-1 and Trans-crocetin. Fitoterapia 92, 290–295. 10.1016/j.fitote.2013.11.014 24321578

[B74] Lautenschläger M. Sendker J. Hüwel S. Galla H. J. Brandt S. Düfer M. (2015). Intestinal Formation of Trans-crocetin from Saffron Extract (Crocus Sativus L.) and *In Vitro* Permeation through Intestinal and Blood Brain Barrier. Phytomedicine 22 (1), 36–44. 10.1016/j.phymed.2014.10.009 25636868

[B75] Lee I. A. Lee J. H. Baek N. I. Kim D. H. (2005). Antihyperlipidemic Effect of Crocin Isolated from the Fructus of Gardenia Jasminoides and its Metabolite Crocetin. Biol. Pharm. Bull. 28 (11), 2106–2110. 10.1248/bpb.28.2106 16272698

[B76] Li C. Y. Huang W. F. Wang Q. L. Wang F. Cai E. Hu B. (2012). Crocetin Induces Cytotoxicity in Colon Cancer Cells via P53-independent Mechanisms. Asian Pac. J. Cancer Prev. 13 (8), 3757–3761. 10.7314/APJCP.2012.13.8.3757 23098467

[B77] Li N. Fan M. Li Y. Qian H. Zhang H. Qi X. (2020). Stability Assessment of Crocetin and Crocetin Derivatives in Gardenia Yellow Pigment and Gardenia Fruit Pomace in Presence of Different Cooking Methods. Food Chem. 312, 126031. 10.1016/j.foodchem.2019.126031 31874411

[B78] Li S. Jiang S. Jiang W. Zhou Y. Shen X. Y. Luo T. (2015). Anticancer Effects of Crocetin in Human Esophageal Squamous Cell Carcinoma KYSE-150 Cells. Oncol. Lett. 9 (3), 1254–1260. 10.3892/ol.2015.2869 25663893 PMC4315057

[B79] Li S. Shen X. Y. Ouyang T. Qu Y. Luo T. Wang H. Q. (2017). Synergistic Anticancer Effect of Combined Crocetin and Cisplatin on KYSE-150 Cells via P53/p21 Pathway. Cancer Cel Int 17 (1), 98. 10.1186/s12935-017-0468-9 PMC5663096 29093644

[B80] Li Y. Kakkar R. Wang J. (2018). *In Vivo* and *In Vitro* Approach to Anti-arthritic and Anti-inflammatory Effect of Crocetin by Alteration of Nuclear Factor-E2-Related Factor 2/hem Oxygenase (HO)-1 and NF-Κb Expression. Front. Pharmacol. 9, 1341. 10.3389/fphar.2018.01341 30618728 PMC6299880

[B81] Liao Z. Q. Hu M. Y. Wang J. (2011). Study on the Antioxidant Effect of Crocetin in Interference of Fatty Liver Cells. Acta Nutrimenta Sinica 33 (002), 167–172. 10.13325/j.cnki.acta.nutr.sin.2011.02.021

[B82] Lin S. Li Q. Jiang S. Xu Z. Jiang Y. Liu L. (2020). Crocetin Ameliorates Chronic Restraint Stress-Induced Depression-like Behaviors in Mice by Regulating MEK/ERK Pathways and Gut Microbiota. J. Ethnopharmacol 268 (1), 113608. 10.1016/j.jep.2020.113608 33242618

[B83] Liu J. Ye C. Lv G. Li G. Gao Y. Ji X. (2021). Crocetin Improves Ischaemic Stroke *In Vitro* and Vivo. Arch. Med. Sci. 10.5114/AOMS/133886

[B84] Liu P. Xue Y. Zheng B. Liang Y. Zhang J. Shi J. (2020a). Crocetin Attenuates the Oxidative Stress, Inflammation and Apoptosisin Arsenic Trioxide-Induced Nephrotoxic Rats: Implication of PI3K/AKT Pathway. Int. Immunopharmacol 88, 106959. 10.1016/j.intimp.2020.106959 32919218

[B85] Liu R. (2019). Protective Effect and Mechanism of Saffronic Acid Modified on Myocardial Ischemia-Reperfusion Injury. master’s thesis. Harbin, China: Harbin University of Commerce.

[B86] Liu T. Qian Z. (2003). Protective Effect of Crocetin on Isoproterenol-Induced Myocardial Injury in Rats. Chin. Traditional Herbal Drugs 34 (5), 439–442.

[B87] Liu T. Z. Qian Z. Y. (2002). Pharmacokinetics of Crocetin in Rats. Yao Xue Xue Bao 37 (5), 367–369. 10.1002/0470855304.oth 12579843

[B88] Liu Y. Liang Y. Zheng B. Chu L. Ma D. Wang H. (2020b). Protective Effects of Crocetin on Arsenic Trioxide-Induced Hepatic Injury: Involvement of Suppression in Oxidative Stress and Inflammation through Activation of Nrf2 Signaling Pathway in Rats. Drug Des. Devel Ther. 14, 1921–1931. 10.2147/DDDT.S247947 PMC7245440 32546959

[B89] Llorens S. Mancini A. Serrano-Díaz J. D'Alessandro A. M. Nava E. Alonso G. L. (2015). Effects of Crocetin Esters and Crocetin from Crocus Sativus L. On Aortic Contractility in Rat Genetic Hypertension. Molecules 20 (9), 17570–17584. 10.3390/molecules200917570 26402666 PMC6332434

[B90] Lou S. Wang L. He L. Wang Z. Wang G. Lin X. (2016). Production of Crocetin in Transgenic Chlorella Vulgaris Expressing Genes crtRB and ZCD1. J. Appl. Phycol 28 (28), 1657–1665. 10.1007/s10811-015-0730-2

[B91] Magesh V. Durgabhavani K. Senthilnathan P. Rajendran P. Sakthisekaran D. (2010). *In Vivo* protective Effect of Crocetin on Benzo(a)pyrene-Induced Lung Cancer in Swiss Albino Mice. Phytother Res. 23 (4), 533–539. 10.1002/ptr.2666 19067387

[B92] Magesh V. Singh J. P. Selvendiran K. Ekambaram G. Sakthisekaran D. (2006). Antitumour Activity of Crocetin in Accordance to Tumor Incidence, Antioxidant Status, Drug Metabolizing Enzymes and Histopathological Studies. Mol. Cel Biochem 287 (1-2), 127–135. 10.1007/s11010-005-9088-0 16685462

[B93] Mahdavifard S. Bathaie S. Z. Nakhjavani M. Taghikhani M. (2016). The Synergistic Effect of Antiglycating Agents (MB-92) on Inhibition of Protein Glycation, Misfolding and Diabetic Complications in Diabetic-Atherosclerotic Rat. Eur. J. Med. Chem. 121, 892–902. 10.1016/j.ejmech.2015.11.035 26733359

[B94] Mahdieh N. Yousef R. Rahbarghazi R. Kheradmand F. Karimipour M. Aramwit P. (2019). Crocetin Promotes Angiogenesis in Human Endothelial Cells through PI3K-Akt-eNOS Signaling Pathway. EXCLI J. 18, 936–949. 10.17179/excli2019-1175 31762720 PMC6868919

[B95] Mancini A. Serrano-Díaz J. Nava E. D'Alessandro A. M. Alonso G. L. Carmona M. (2014). Crocetin, a Carotenoid Derived from Saffron (Crocus Sativus L.), Improves Acetylcholine-Induced Vascular Relaxation in Hypertension. J. Vasc. Res. 51 (5), 393–404. 10.1159/000368930 25531977

[B96] Martin G. Goh E. Neff A. W. (2002). Evaluation of the Developmental Toxicity of Crocetin on Xenopus. Food Chem. Toxicol. 40 (7), 959–964. 10.1016/S0278-6915(02)00040-6 12065218

[B97] Maysam S. S. Zahra B. S. Heydarzade H. (2011). Effect of Crocin and Crocetin on EDA Activity in NMU-Induced Breast Cancer in Rat. Clin. Biochem. 44 (13), S27. 10.1016/J.CLINBIOCHEM.2011.08.080

[B98] Meng L. Cui L. (2008). Inhibitory Effects of Crocetin on High Glucose-Induced Apoptosis in Cultured Human Umbilical Vein Endothelial Cells and its Mechanism. Arch. Pharm. Res. 31, 357–363. 10.1007/s12272-001-1164-y 18409050

[B99] Mertes P. M. Collange. Collange O. Coliat P. Banerjee M. Diringer M. C. Roche A. (2021). Liposomal Encapsulation of Trans-crocetin Enhances Oxygenation in Patients with COVID-19-Related ARDS Receiving Mechanical Ventilation. J. Control. Release 336, 252–261. 10.1016/j.jconrel.2021.06.033 34175365 PMC8225316

[B100] Mh A. Hhb C. (2019). A Comprehensive Review on Biological Activities and Toxicology of Crocetin. Food Chem. Toxicol. 130, 44–60. 31100302 10.1016/j.fct.2019.05.017

[B101] Michael C. P. Derpapas M. Aravidou E. Sofopoulos M. Michael P. Polydorou A. (2020). The Carotenoid Compound of Saffron Crocetin Alleviates Effects of Ischemia Reperfusion Injury via a Mechanism Possibly Involving MiR-127. Cureus 12 (2), e6979. 10.7759/cureus.6979 32089976 PMC7017928

[B102] Milajerdi A. Djafarian K. Hosseini B. (2016). The Toxicity of Saffron (Crocus Sativus L.) and its Constituents against normal and Cancer Cells. J. Nutr. Intermediary Metab. 3, 23–32. 10.1016/j.jnim.2015.12.332

[B103] Miller T. L. Willett S. L. Moss M. E. Miller J. Belinka B. A. (1982). Binding of Crocetin to Plasma Albumin. J. Pharm. Sci. 71 (2), 173–177. 10.1002/JPS.2600710209 7062239

[B104] Mir M. A. Ganai S. A. Mansoor S. Jan S. Mani P. Masoodi K. Z. (2020). Isolation, Purification and Characterization of Naturally Derived Crocetin Beta-D-Glucosyl Ester from Crocus Sativus L. Against Breast Cancer and its Binding Chemistry with ER-alpha/HDAC2. Saudi J. Biol. Sci. 27 (3), 975–984. 10.1016/j.sjbs.2020.01.018 32127777 PMC7042633

[B105] Modenutti C. P. Blanco Capurro J. I. Ibba R. Vasiljević S. Hensen M. Alonzi D. S. (2019). Clamping, Bending, and Twisting Inter-domain Motions in the Misfold-Recognising Portion of UDP-Glucose:glycoprotein Glucosyl-Transferase. Structure 29, 357–370. 10.1101/2019.12.25.888438 PMC8024514 33352114

[B106] Mohan C. D. Kim C. Siveen K. S. Manu K. A. Rangappa S. Chinnathambi A. (2021). Crocetin Imparts Antiproliferative Activity via Inhibiting STAT3 Signaling in Hepatocellular Carcinoma. IUBMB life 73, 1348–1362. 10.1002/iub.2555 34514729

[B107] Moradzadeh M. Ghorbani A. Erfanian S. Mohaddes S. T. Rahimi H. karimiani E. G. (2019). Study of the Mechanisms of Crocetin‐induced Differentiation and Apoptosis in Human Acute Promyelocytic Leukemia Cells. J. Cel Biochem 120, 1943–1957. 10.1002/jcb.27489 30203596

[B108] Moraga A. R. Nohales P. F. Pérez J. A. Gómez-Gómez L. (2004). Glucosylation of the Saffron Apocarotenoid Crocetin by a Glucosyltransferase Isolated from Crocus Sativus Stigmas. Planta 219 (6), 955–966. 10.1007/s00425-004-1299-1 15605174

[B109] Mori K. Torii H. Fujimoto S. Jiang X. Ikeda S. I. Yotsukura E. (2019). The Effect of Dietary Supplementation of Crocetin for Myopia Control in Children: A Randomized Clinical Trial. J. Clin. Med. 8 (8), 1179. 10.3390/jcm8081179 PMC6724222 31394821

[B110] Murphy E. Steenbergen C. (2008). Mechanisms Underlying Acute Protection from Cardiac Ischemia-Reperfusion Injury. Physiol. Rev. 88 (2), 581–609. 10.1152/physrev.00024.2007 18391174 PMC3199571

[B111] Németh K. Plumb G. W. Berrin J. G. Juge N. Jacob R. Naim H. Y. (2003). Deglycosylation by Small Intestinal Epithelial Cell Beta-Glucosidases Is a Critical Step in the Absorption and Metabolism of Dietary Flavonoid Glycosides in Humans. Eur. J. Nutr. 42 (1), 29–42. 10.1007/s00394-003-0397-3 12594539

[B112] Neyshaburinezhad N. Kalalinia F. Hashemi M. (2019). Encapsulation of Crocetin into Poly (Lactic-co-glycolic Acid) Nanoparticles Overcomes Drug Resistance in Human Ovarian Cisplatin-Resistant Carcinoma Cell Line (A2780-RCIS). Mol. Biol. Rep. 46, 6525–6532. 10.1007/s11033-019-05098-7 31646427

[B113] Nitta K. Nishinaka A. Hida Y. Nakamura S. Shimazawa M. Hara H. (2019). Oral and Ocular Administration of Crocetin Prevents Retinal Edema in a Murine Retinal Vein Occlusion Model. Mol. Vis. 25, 859–868. 31908404 PMC6937220

[B114] Ohno Y. Nakanishi T. Umigai N. Tsuruma K. Shimazawa M. Hara H. (2012). Oral Administration of Crocetin Prevents Inner Retinal Damage Induced by N-Methyl-D-Aspartate in Mice. Eur. J. Pharmacol. 690 (1-3), 84–89. 10.1016/j.ejphar.2012.06.035 22760072

[B115] Oliveira H. Cai X. Zhang Q. de Freitas V. Mateus N. He J. (2017). Gastrointestinal Absorption, Antiproliferative and Anti-inflammatory Effect of the Major Carotenoids of Gardenia Jasminoides Ellis on Cancer Cells. Food Funct. 8 (4), 1672–1679. 10.1039/c7fo00091j 28322405

[B116] Parizadeh M. Gharib F. G. Abbaspour A. Afshar T. Ghayour M. (2011). Effects of Aqueous Saffron Extract on Nitric Oxide Production by Two Human Carcinoma Cell Lines: Hepatocellular Carcinoma (HepG2) and Laryngeal Carcinoma (Hep2). avicenna J. phytomedicine 1, 43–50. 10.22038/AJP.2011.120

[B117] Patel N. K. Bhutani K. K. (2014). Suppressive Effects of Mimosa Pudica (L.) Constituents on the Production of LPS-Induced Pro-inflammatory Mediators. EXCLI J. 13, 1011–1021. 26417317 PMC4464187

[B118] Peng F. C. Gao C. Qian Z. Y. (2007). Protective Effects of Crocetin on Anoxic Injury in Mice. Chin. J. New Drugs 16 (21), 1772–1775.

[B119] Peng M. Li-Juan M. A. Sun Z. W. Wang Y. H. Wang X. E. (2019). Changes of Inflammatory Factors and T Lymphocyte Subsets in Patients with Cervical Cancer after Concurrent Radiotherapy and Chemotherapy. Chin. J. Nosocomiology 29 (19), 3022–3026.

[B120] Pradhan J. Mohanty C. Sahoo S. K. (2018). Protective Efficacy of Crocetin and its Nanoformulation against Cyclosporine A-Mediated Toxicity in Human Embryonic Kidney Cells. Life Sci. 216, 39–48. 10.1016/j.lfs.2018.11.027 30444987

[B121] Puglia C. Santonocito D. Musumeci T. Cardile V. Graziano A. Salerno L. (2018). Nanotechnological Approach to Increase the Antioxidant and Cytotoxic Efficacy of Crocin and Crocetin. Planta Med. 85, 258–265. 10.1055/a-0732-5757 30206907

[B122] Qian H. Zhao B. T. Xu D. R. Huang X. D. (2010). Preparation of Crocetin from Gadenia Yellow Pigment. CHINESE WILD PLANT RESOURCES 29 (05), 26–28. 10.3969/j.issn.1006-9690.2010.05.007

[B123] Qin L. Liu H. Wang J. Wang W. Zhang L. (2021). Crocetin Exerts a Cardio-Protective Effect on Mice with Coxsackievirus B3-Induced Acute Viral Myocarditis. J. Oleo Sci. 70 (8), 1115–1124. 10.5650/jos.ess21100 34349088

[B124] Rangarajan P. Subramaniam D. Paul S. Kwatra D. Palaniyandi K. Islam S. (2015). Crocetinic Acid Inhibits Hedgehog Signaling to Inhibit Pancreatic Cancer Stem Cells. Oncotarget 6 (29), 27661–27673. 10.18632/oncotarget.4871 26317547 PMC4695016

[B125] Rangarajan P. Subramaniam D. Paul S. Kwatra D. Palaniyandi K. Islam S. (2015). Crocetinic Acid Inhibits Hedgehog Signaling to Inhibit Pancreatic Cancer Stem Cells. Oncotarget 6, 27661–27673. 10.18632/oncotarget.4871 26317547 PMC4695016

[B126] Ray P. Guha D. Chakraborty J. Banerjee S. Adhikary A. Chakraborty S. (2016). Crocetin Exploits P53-Induced Death Domain (PIDD) and FAS-Associated Death Domain (FADD) Proteins to Induce Apoptosis in Colorectal Cancer. Sci. Rep. 6, 32979. 10.1038/srep32979 27622714 PMC5020693

[B127] Razavi B. M. Hosseinzadeh H. (2015). Saffron as an Antidote or a Protective Agent against Natural or Chemical Toxicities. Daru 23 (1), 31–39. 10.1186/s40199-015-0112-y 25928729 PMC4418072

[B128] Reddy C. N. Bharate S. B. Vishwakarma R. A. Bharate S. S. (2020). Chemical Analysis of Saffron by HPLC Based Crocetin Estimation. J. Pharm. Biomed. Anal. 181, 113094. 10.1016/j.jpba.2020.113094 31927167

[B129] Sazgarnia A. Salarabadi S. S. Hashemi M. (2021). The Role of Crocetin-Loaded PLGA Nanoparticles as a Pre-treatment Agent on Indocyanine-Photodynamic Therapy of Breast Cancer Cells. Iranian J. Med. Phys. 10.22038/IJMP.2021.56373.1942

[B130] Schulz-Schaeffer W. J. (2010). The Synaptic Pathology of Alpha-Synuclein Aggregation in Dementia with Lewy Bodies, Parkinson's Disease and Parkinson's Disease Dementia. Acta Neuropathol. 120 (2), 131–143. 10.1007/s00401-010-0711-0 20563819 PMC2892607

[B131] Sepahi S. Soheili Z.-S. Tavakkol-Afshari J. Mehri S. Hosseini S. M. Mohajeri S. A. (2021). Retinoprotective Effects of Crocin and Crocetin via Anti-angiogenic Mechanism in High Glucose-Induced Human Retinal Pigment Epithelium Cells. Cmp 14, 883–893. 10.2174/1874467214666210420111232 33881975

[B132] Shen X. C. Qian Z. Y. (2006). Effects of Crocetin on Antioxidant Enzymatic Activities in Cardiac Hypertrophy Induced by Norepinephrine in Rats. Pharmazie 61 (4), 348–352. 10.1080/14786410500185584 16649553

[B133] Sheng L. Qian Z. Shi Y. Yang L. Xi L. Zhao B. (2008). Crocetin Improves the Insulin Resistance Induced by High-Fat Diet in Rats. Br. J. Pharmacol. 154 (5), 1016–1024. 10.1038/bjp.2008.160 18469847 PMC2451043

[B134] Shi L. Xie G. Y. Wang S. Meng Y. Qin M. J. (2016). Advance in Pharmaceutical Research of Buddleia Officinalis Maxim. Chin. Wild Plant Resour. 35 (03), 34–40.

[B135] Song T. Wu N. Wang C. Wang Y. Chai F. Ding M. (2020). Crocetin Overproduction in Engineered *Saccharomyces cerevisiae* via Tuning Key Enzymes Coupled with Precursor Engineering. Front. Bioeng. Biotechnol. 8, 578005. 10.3389/fbioe.2020.578005 33015027 PMC7500066

[B136] Song Y. Zhu L. Li M. (2013). Antifibrotic Effects of Crocetin in Scleroderma Fibroblasts and in Bleomycin-Induced Sclerotic Mice. Clinics (Sao Paulo) 68 (10), 1350–1357. 10.6061/clinics/2013(10)10 24212843 PMC3798612

[B137] Sosa V. Moliné T. Somoza R. Paciucci R. Kondoh H. LLeonart M. E. (2012). Oxidative Stress and Cancer: An Overview. Ageing Res. Rev. 12 (1), 376–390. 10.1016/j.arr.2012.10.004 23123177

[B138] Sreekanth G. P. Chuncharunee A. Yenchitsomanus P. T. Limjindaporn T. (2020). Crocetin Improves Dengue Virus-Induced Liver Injury. Viruses 12 (8), 825. 10.3390/v12080825 PMC7472398 32751420

[B139] Sujata V. Ravishankar G. A. Venkataraman L. V. (1992). Methods for the Analysis of the Saffron Metabolites Crocin, Crocetins, Picrocrocin and Safranal for the Determination of the Quality of the Spice Using Thin-Layer Chromatography, High-Performance Liquid Chromatography and Gas Chromatography. J. Chromatogr. A 624 (1–2), 497–502. 10.1016/0021-9673(92)85699-T

[B140] Sun P. D. Wang J. X. Fang Z. K. Tang D. D. (2012). Synthesis of Crocetin Dimethyl Ester with Wittig and Wittig-Horner Reaction. Chem. World 53 (06), 353–357. 10.19500/j.cnki.0367-6358.2012.06.010

[B141] Tahereh F. Saeed S. (2014). The Effect of Saffron (Crocus Sativus L.) and its Ingredients on the Management of Diabetes Mellitus and Dislipidemia. Afr. J. Pharm. Pharmacol. 8 (20), 541–549. 10.5897/AJPPX2013.0006

[B142] Tan A. X. Li X. Y. (2012). Effect of Crocetin on Expression of caspase-3mRNA and NF-Κb in Cerebral Ischemia-Reperfusion of Rats. Chin. J. Hosp. Pharm. 32 (1), 8–11. 10.13286/j.cnki.chinhosppharmacyj.2012.01.002

[B143] Tan A. X. Zhu Y. B. Wang Y. Y. (2011). Effect of Crocetin on Free Radicals and Nitrogen Monoxidum during Reperfusion after Cerebral Ischemia in Rats. Herald Med. 30 (07), 846–848. 10.3870/yydb.2011.07.004

[B144] Tan H. Chen X. Liang N. Chen R. Chen J. Hu C. (2019). Transcriptome Analysis Reveals Novel Enzymes for Apo-Carotenoid Biosynthesis in Saffron and Allows Construction of a Pathway for Crocetin Synthesis in Yeast. J. Exp. Bot. 70 (18), 4819–4834. 10.1093/jxb/erz211 31056664

[B145] Tang Y. Lou Z. Yang L. Wang H. (2015). Screening of Antimicrobial Compounds against Salmonellaty Phimurium from Burdock (Arctium Lappa) Leaf Based on Metabolomics. Eur. Food Res. Technol. 240 (6), 1203–1209. 10.1007/s00217-015-2423-0

[B146] Tarantilis P. A. Morjani H. Polissiou M. Manfait M. (1994). Inhibition of Growth and Induction of Differentiation of Promyelocytic Leukemia (HL-60) by Carotenoids from Crocus Sativus L. Anticancer Res. 14 (5A), 1913–1918. 7847826

[B147] Tashakori-Sabzevar F. Hosseinzadeh H. Motamedshariaty V. S. Movassaghi A. R. Mohajeri S. A. (2013). Crocetin Attenuates Spatial Learning Dysfunction and Hippocampal Injury in a Model of Vascular Dementia. Curr. Neurovasc Res. 10 (4), 325–334. 10.2174/15672026113109990032 23988025

[B148] Tiribuzi R. Crispoltoni L. Chiurchiù V. Casella A. Montecchiani C. Del Pino A. M. (2016). Trans-crocetin Improves Amyloid-β Degradation in Monocytes from Alzheimer's Disease Patients. J. Neurol. Sci. 372, 408–412. 10.1016/j.jns.2016.11.004 27865556

[B149] Tsantarliotou M. P. Poutahidis T. Markala D. Kazakos G. Sapanidou V. Lavrentiadou S. (2013). Crocetin Administration Ameliorates Endotoxin-Induced Disseminated Intravascular Coagulation in Rabbits. Blood Coagul. Fibrinolysis 24 (3), 305–310. 10.1097/MBC.0b013e32835bdc8f 23358225

[B150] Umigai N. Murakami K. Ulit M. V. Antonio L. S. Shirotori M. Morikawa H. (2011). The Pharmacokinetic Profile of Crocetin in Healthy Adult Human Volunteers after a Single Oral Administration. Phytomedicine 18 (7), 575–578. 10.1016/j.phymed.2010.10.019 21112749

[B151] Umigai N. Takeda R. Mori A. (2018). Effect of Crocetin on Quality of Sleep: A Randomized, Double-Blind, Placebo-Controlled, Crossover Study. Complement. Ther. Med. 41, 47–51. 10.1016/j.ctim.2018.09.003 30477864

[B152] Umigai N. Takeda R. Mori A. (2018). Effect of Crocetin on Quality of Sleep: A Randomized, Double-Blind, Placebo-Controlled, Crossover Study. Complement. Therapies Med. 41, 47–51. 10.1016/j.ctim.2018.09.003 30477864

[B153] Wang C. J. Lee M. J. Chang M. C. Lin J. K. (1995). Inhibition of Tumor Promotion in Benzo[a]pyrene-Initiated CD-1 Mouse Skin by Crocetin. Carcinogenesis 16 (2), 187–191. 10.1093/carcin/16.2.187 7859347

[B154] Wang C. J. Shiow S. J. Lin J. K. (1991). Effects of Crocetin on the Hepatotoxicity and Hepatic DNA Binding of Aflatoxin B1 in Rats. Carcinogenesis 12 (03), 459–462. 10.1093/CARCIN/12.3.459 1672627

[B155] Wang F. X. Wang Y. Mei X. Q. Yang P. Q. Wang Y. Y. (2017a). An Experimental Study on the Treatment of Liver Fibrosis with Crocetin. Prog. Mod. Biomed. (28), 5432–5435. 10.13241/j.cnki.pmb.2017.28.007

[B156] Wang H. D. Li l. (2015). Crocetin Salt Injection and Preparation Process Thereof.

[B157] Wang H. F. Ma J. X. Shang Q. L. An J. B. Chen H. T. Wang C. X. (2018a). Safety, Pharmacokinetics, and Prevention Effect of Intraocular Crocetin in Proliferative Vitreoretinopathy. Biomed. Pharmacother. 109, 1211–1220. 10.1016/j.biopha.2018.10.193 30551371

[B158] Wang H. F. (2018). The Inhibition Effect and Molecular Mechanism of Crocetin on Development of Proliferative Vitreoretinopathy. Dissertation’s thesis. Shijiazhuang, China: Hebei Medical University.

[B159] Wang M. Z. Gao J. Chu Y. Niu J. Chen M. Shang Q. (2020a). Synthesis of Crocetin Derivatives and Their Potent Inhibition in Multiple Tumor Cells Proliferation and Inflammatory Property of Macrophage. BMC Complement. Med. Ther. 20 (1), 1–8. 10.1186/s12906-020-2831-y 32020873 PMC7076819

[B160] Wang R. Shen G. P. (2012). Effect of Different Concentrations of Crocetin on the Expression of HERG Potassium Channel Protein. Chin. high Alt. Med. Biol. 33 (3), 149–152.

[B161] Wang S. L. Huang D. Yang F. Y. Huang W. (2018b). Absorption and Transport of Crocetin in Caco-2 Cell Model. Chin. J. Clin. Pharmacol. 34 (15), 1894–1897. 10.13699/j.cnki.1001-6821.2018.15.045

[B162] Wang X. Zhang G. Qiao Y. Feng C. Zhao X. (2017b). Crocetin Attenuates Spared Nerve Injury-Induced Neuropathic Pain in Mice. J. Pharmacol. Sci. 135 (4), 141–147. 10.1016/j.jphs.2017.08.007 29217355

[B163] Wang Y. Sun J. Liu C. Fang C. (2014). Protective Effects of Crocetin Pretreatment on Myocardial Injury in an Ischemia/reperfusion Rat Model. Eur. J. Pharmacol. 741, 290–296. 10.1016/j.ejphar.2014.07.052 25176181

[B164] Wang Y. Yan J. Xi L. Qian Z. Wang Z. Yang L. (2012). Protective Effect of Crocetin on Hemorrhagic Shock-Induced Acute Renal Failure in Rats. Shock 38 (1), 63–67. 10.1097/SHK.0b013e3182596ec4 22576007

[B165] Wang Y. Yu W. Shi C. Hu P. (2020b). Crocetin Attenuates Sepsis-Induced Cardiac Dysfunction via Regulation of Inflammatory Response and Mitochondrial Function. Front. Physiol. 11. 10.3389/fphys.2020.00514 PMC7295980 32581829

[B166] Wani A. Al Rihani S. B. Sharma A. Weadick B. Govindarajan R. Khan S. U. (2021). Crocetin Promotes Clearance of Amyloid-β by Inducing Autophagy via the STK11/LKB1-Mediated AMPK Pathway. Autophagy 17, 3813–3832. 10.1080/15548627.2021.1872187 33404280 PMC8632093

[B167] Wen N. Qian Z. Y. Rao S. Y. Shen Y. C. (2005). Effects of Crocetin on Energy Metabolism in Rats with Myocardial Ischemia-Reperfusion Injury. Chin. J. New Drugs 14 (11), 4. 10.3321/j.issn:1003-3734.2005.11.013

[B168] Wen Y.-L. He Z. Hou D.-X. Qin S. (2021). Crocetin Exerts its Anti-inflammatory Property in LPS-Induced RAW264.7 Cells Potentially via Modulation on the Crosstalk between MEK1/JNK/NF-κB/iNOS Pathway and Nrf2/HO-1 Pathway. Oxidative Med. Cell Longevity 2021, 1–18. 10.1155/2021/6631929 PMC8449229 34545298

[B169] Wong K. H. Xie Y. Huang X. Kadota K. Yao X. S. Yu Y. (2020). Delivering Crocetin across the Blood-Brain Barrier by Using γ-Cyclodextrin to Treat Alzheimer's Disease. Sci. Rep. 10 (1), 3654. 10.1038/s41598-020-60293-y 32107408 PMC7046745

[B170] Wüthrich B. Schmid-Grendelmeyer P. Lundberg M. (2010). Anaphylaxis to Saffron. Allergy 52 (4), 476–477. 10.1111/j.1398-9995.1997.tb01034.x 9188936

[B171] Xi L. Qian Z. Xu G. Zheng S. Sun S. Wen N. (2007). Beneficial Impact of Crocetin, a Carotenoid from Saffron, on Insulin Sensitivity in Fructose-Fed Rats. J. Nutr. Biochem. 18 (01), 64–72. 10.1016/J.JNUTBIO.2006.03.010 16713230

[B172] Xi L. Qian Z. (2006). Pharmacological Properties of Crocetin and Crocin (Digentiobiosyl Ester of Crocetin) from Saffron. Nat. Prod. Commun. 1 (1), 1934578X0600100–75. 10.1177/1934578X0600100112

[B173] Xia S. Peng Y. Jia Q. Lin H. (2018). Research Process on Saffron Glycosides from Gardenia Jasminoides. South China For. Sci. 46 (06), 51–54. 10.16259/j.cnki.36-1342/s.2018.06.012

[B174] Xiang M. Yang R. Zhang Y. Wu P. Wang L. Gao Z. (2017). Effect of Crocetin on Vascular Smooth Muscle Cells Migration Induced by Advanced Glycosylation End Products. Microvasc. Res. 112, 30–36. 10.1016/j.mvr.2017.02.004 28209519

[B175] Xiang M. Qian Z. Y. Zhou C. H. (2006). Effects of Crocetin on Formation of Advanced Glycation End Products and Expression of Releptor for Advanced Glycation and Prodmts Protein in Diabetic Rats. Chin. J. Clin. Pharmacol. Ther. 11 (4), 448–452. 10.3969/j.issn.1009-2501.2006.04.021

[B176] Xiao W. H. Mei X. A. Chen Y. Wang Y. Yuan Y. J. (2019). An Engineering Bacterium and its Construction Method and Application in the Preparation of Crocetin.

[B177] Xu M. M. (2019). The Role of microRNA-27a in Ang Ⅱ-induced Proliferation and Migration of Vascular Smooth Muscle Cells and its Mechanism. master’s thesis. Dalian, China: Dalian Medical University.

[B178] Yamauchi M. Tsuruma K. Imai S. Nakanishi T. Umigai N. Shimazawa M. (2011). Crocetin Prevents Retinal Degeneration Induced by Oxidative and Endoplasmic Reticulum Stresses via Inhibition of Caspase Activity. Eur. J. Pharmacol. 650 (1), 110–119. 10.1016/j.ejphar.2010.09.081 20951131

[B179] Yan J. Qian Z. Sheng L. Zhao B. Yang L. Ji H. (2010). Effect of Crocetin on Blood Pressure Restoration and Synthesis of Inflammatory Mediators in Heart after Hemorrhagic Shock in Anesthetized Rats. Shock 33 (1), 83–87. 10.1097/SHK.0b013e3181a98f55 19487985

[B180] Yang L. Qian Z. Ji H. Yang R. Wang Y. Xi L. (2010). Inhibitory Effect on Protein Kinase Ctheta by Crocetin Attenuates Palmitate-Induced Insulin Insensitivity in 3T3-L1 Adipocytes. Eur. J. Pharmacol. 642 (1-3), 47–55. 10.1016/j.ejphar.2010.05.061 20541543

[B181] Yang L. Qian Z. Yang Y. Sheng L. Ji H. Zhou C. (2008). Involvement of Ca2+ in the Inhibition by Crocetin of Platelet Activity and Thrombosis Formation. J. Agric. Food Chem. 56 (20), 9429–9433. 10.1021/jf802027a 18817408

[B182] Yang X. (2019). Design and Optimization of Crocetin Loaded PLGA Nanoparticles against Diabetic Nephropathy via Suppression of Inflammatory Biomarkers: a Formulation Approach to Preclinical Study. Drug Deliv. 26 (1), 849–859. 10.1080/10717544.2019.1642417 31524015 PMC6761602

[B183] Yang X. Z. Tang C. P. (2008). Chemical Constituents of Stemona Japonica. Nat. Product. Res. Development 020 (003), 399–402. 10.1055/s-0029-1185868

[B184] Yang Y. A. (2012). Crocetin Diammonium Salt.

[B185] Yang Y. G. Zhu H. L. Ji H. Wang X. L. Tang J. F. (2011). Crocetin Organic Amine Salt and its Preparation Method.

[B186] Yao X. S. Zhang D. Yu Y. (2018). Application of Saffron Pigment Composition in the Preparation of Drugs for the Treatment of Parkinson's Disease.

[B187] Yoshino F. Yoshida A. Umigai N. Kubo K. Lee M. C. (2011). Crocetin Reduces the Oxidative Stress Induced Reactive Oxygen Species in the Stroke-Prone Spontaneously Hypertensive Rats (SHRSPs) Brain. J. Clin. Biochem. Nutr. 49 (3), 182–187. 10.3164/jcbn.11-01 22128217 PMC3208014

[B188] Yoshino Y. Ishisaka M. Umigai N. Shimazawa M. Tsuruma K. Hara H. (2014). Crocetin Prevents Amyloid β1-42-Induced Cell Death in Murine Hippocampal Cells. Pp 05 (1), 37–42. 10.4236/pp.2014.51007

[B189] Yu L. Gao R. Song X. Li X. Zhu J. (2021). Cardio-protective and Anti-atherosclerosis Effect of Crocetin on Vitamin D3 and HFD-Induced Atherosclerosis in Rats. J. Oleo Sci. 70 (10), 1447–1459. 10.5650/jos.ess21168 34615830

[B190] Zang M. Hou J. Huang Y. Wang J. Ding X. Zhang B. (2021). Crocetin Suppresses Angiogenesis and Metastasis through Inhibiting Sonic Hedgehog Signaling Pathway in Gastric Cancer. Biochem. Biophys. Res. Commun. 576, 86–92. 10.1016/j.bbrc.2021.08.092 34482028

[B191] Zhang A. Li J. (2017). Crocetin Shifts Autophagic Cell Survival to Death of Breast Cancer Cells in Chemotherapy. Tumour Biol. 39 (3), 1010428317694536. 10.1177/1010428317694536 28351329

[B192] Zhang A. J. Luo J. (2016). Studies on the Synthesis of Crocetin Dialdehyde. Chem. Res. Appl. 28 (08), 1155–1159.

[B193] Zhang H. Shang Q. An J. Wang C. Ma J. (2019a). Crocetin Inhibits PDGF-BB-Induced Proliferation and Migration of Retinal Pigment Epithelial Cells. Eur. J. Pharmacol. 842, 329–337. 10.1016/j.ejphar.2018.11.001 30395849

[B194] Zhang J. Wang Y. Dong X. Liu J. (2018). Crocetin Attenuates Inflammation and Amyloid-β Accumulation in APPsw Transgenic Mice. Immun. Ageing 15, 24. 10.1186/s12979-018-0132-9 30450117 PMC6208089

[B195] Zhang L. H. Zhang G. Z. Liu K. Q. Rong J. D. Zheng Y. S. (2013). Advances in the Research on Resources Development and Utilization of Gardenia Jasminoides Ellis. Subtropical Agric. Res. 9 (04), 231–234. 10.13321/j.cnki.subtrop.agric.res.2013.04.013

[B196] Zhang Q. C. (2020). Investigation on Bioactivities and Mechanism of Crocetin on SGC7901 Cells of Gastric Cancer. master’s thesis. Cnki: Zunyi Medical University.

[B197] Zhang W. Li Y. Ge Z. (2017a). Cardiaprotective Effect of Crocetin by Attenuating Apoptosis in Isoproterenol Induced Myocardial Infarction Rat Model. Biomed. Pharmacother. 93, 376–382. 10.1016/j.biopha.2017.06.032 28651239

[B198] Zhang W. (2017). Research on Preparation of High Purity Crocetin Sodium and its Bioavailability. master’s thesis. Hangzhou, China: Zhejiang Chinese Medical University.

[B199] Zhang X. T. Ma S. W. Wang L. He S. J. Yu J. Y. 2011. The Preparation Method of Crocetin Injection.

[B200] Zhang X. Yan K. Q. Feng D. Q. Ling B. (2019b). Advances in Role of Cyclooxygenase 2 in Development and Progression of Cancer. Cancer Res. Prev. Treat. 46 (11), 1036–1039. 10.3971/j.issn.1000-8578.2019.19.0413

[B201] Zhang Y. Geng J. Hong Y. Jiao L. Li S. Sun R. (2019c). Orally Administered Crocin Protects against Cerebral Ischemia/Reperfusion Injury through the Metabolic Transformation of Crocetin by Gut Microbiota. Front. Pharmacol. 10, 440. 10.3389/fphar.2019.00440 31114499 PMC6502977

[B202] Zhang Y. Fei F. Zhen L. Zhu X. Wang J. Li S. (2017). Sensitive Analysis and Simultaneous Assessment of Pharmacokinetic Properties of Crocin and Crocetin after Oral Administration in Rats. J. Chromatogr. B 1044-1045, 1–7. 10.1016/j.jchromb.2016.12.003 28056427

[B203] Zhang Y. L. Zhang X. Y. Kou Y. F. (2017b). Preparation Technology of Crocetin from Gardenia Jasminoides. Med. Res. Education 34 (4), 21–25. 10.3969/j.issn.1674-490X.2017.04.005

[B204] Zhao Y. J. Lu Y. You Z. P. (2020a). Protective Effect of Crocetin on Retinal Neuroepithelial in Streptozotocin Induced Diabetic Rat. Chin. Pharmacol. Bull. 36 (03), 399–403. 10.3969/j.issn.1001-1978.2020.03.019

[B205] Zhao Z. Zheng B. Li J. Wei Z. Chu S. Han X. (2020b). Influence of Crocetin, a Natural Carotenoid Dicarboxylic Acid in Saffron, on L-type Ca2+ Current, Intracellular Ca2+ Handling and Contraction of Isolated Rat Cardiomyocytes. Biol. Pharm. Bull. 43 (9), 1367–1374. 10.1248/bpb.b20-00298 32879211

[B206] Zhao Z. D. (2015). Red Gold" Saffron. Oriental Medicated Diet. 000 (9), 41–42.

[B207] Zheng S. G. Qian Z. Y. Wang H. T. (2009). Effects of Crocetin on the Susceptibility of LDL to Oxidation and Serum Level of Ox-LDL in Hyperlipidemic Rabbits. Chin. J. Exp. Traditional Med. Formulae 15 (006), 50–53. 10.13422/j.cnki.syfjx.2009.06.022

[B208] Zheng Y. Zhu N. Wang J. Zhao N. Yuan C. (2021). Crocetin Suppresses Gestational Diabetes in Streptozotocin‐induced Diabetes Mellitus Rats via Suppression of Inflammatory Reaction. J. Food Biochem. 45 (9), e13857. 10.1111/jfbc.13857 34309046

[B209] Zhong H. (2014). Study on Solid Dispersion Sustained Release Tablets of Crocetin. master’s thesis. Jiangsu, China: Jiangsu University.

[B210] Zhong Y. J. Shi F. Zheng X. L. Wang Q. Yang L. Sun H. (2011). Crocetin Induces Cytotoxicity and Enhances Vincristine-Induced Cancer Cell Death via P53-dependent and -independent Mechanisms. Acta Pharmacol. Sin 32, 1529–1536. 10.1038/aps.2011.109 21986580 PMC4010206

[B211] Zhou C. H. Qian Z. Y. Zheng S. G. Xiang M. (2006). ERK1/2 Pathway Is Involved in the Inhibitory Effect of Crocetin on Angiotensin II-Induced Vascular Smooth Muscle Cell Proliferation. Eur. J. Pharmacol. 535 (1-3), 61–68. 10.1016/j.ejphar.2006.02.027 16580346

[B212] Zhou C. H. Xiang M. He S. Y. Qian Z. Y. (2010). Crocetin Inhibits Cell Cycle G1/S Transition through Suppressing Cyclin D1 and Elevating P27kip1 in Vascular Smooth Muscle Cells. Phytother Res. 24, 975–981. 10.1002/ptr.3039 20041429

[B213] Zhou H. Yuan X. Zhao Q. Zhao B. Wang X. (2013). Determination of Oxygen Transmission Barrier of Microcapsule wall by Crocetin Deterioration Kinetics. Eur. Food Res. Technol. 237 (4), 639–646. 10.1007/s00217-013-2022-x

[B214] Zhu H. L. Wang X. L. Tang J. F. Yang Y. S. (2012). Preparation Method and Application of a Kind of Crocetin Amide Derivatives.

[B215] Zhuang X. Dong A. Wang R. Shi A. (2018). Crocetin Treatment Inhibits Proliferation of colon Cancer Cells through Down-Regulation of Genes Involved in the Inflammation. Saudi J. Biol. Sci. 25 (8), 1767–1771. 10.1016/j.sjbs.2017.04.005 30591798 PMC6303136

[B216] Zou Z. Chang H. Li H. Wang S. (2017). Induction of Reactive Oxygen Species: an Emerging Approach for Cancer Therapy. Apoptosis 22, 1321–1335. 10.1007/s10495-017-1424-9 28936716

